# Identification of host genetic factors modulating β-lactam resistance in *Escherichia coli* harbouring plasmid-borne β-lactamase through transposon-sequencing

**DOI:** 10.1080/22221751.2025.2493921

**Published:** 2025-04-15

**Authors:** Hyunhee Kim, Travis Bell, Kihyun Lee, Jeongyun Jeong, James C. A. Bardwell, Changhan Lee

**Affiliations:** a Department of Biological Sciences, Ajou University, Suwon, South Korea; b Research Institute of Basic Sciences, Ajou University, Suwon, South Korea; c Department of Molecular, Cellular, and Developmental Biology, University of Michigan, Ann Arbor, MI, USA; d CJ Bioscience, Seoul, South Korea; e Howard Hughes Medical Institute, University of Michigan, Ann Arbor, MI, USA

**Keywords:** β-lactamase producing bacteria, β-lactam resistance, *Escherichia coli*, *Pseudomonas aeruginosa*, *skp*, *gshA*, *phoPQ*, *ypfN*

## Abstract

Since β-lactam antibiotics are widely used, emergence of bacteria with resistance to them poses a significant threat to society. In particular, acquisition of genes encoding β-lactamase, an enzyme that degrades β-lactam antibiotics, has been a major contributing factor in the emergence of bacteria that are resistant to β-lactam antibiotics. However, relatively few genetic targets for killing these resistant bacteria have been identified to date. Here, we used a systematic approach called transposon-sequencing (Tn-Seq), to screen the *Escherichia coli* genome for host genetic factors that, when mutated, affect resistance to ampicillin, one of the β-lactam antibiotics, in a strain carrying a plasmid that encodes β-lactamase. This approach enabled not just the isolation of genes previously known to affect β-lactam resistance, but the additional loci *skp*, *gshA*, *phoPQ* and *ypfN*. Individual mutations in these genes modestly but consistently affected antibiotic resistance. We have identified that these genes are not only implicated in β-lactam resistance by itself but also play a crucial role in conditions associated with the expression of β-lactamase. *GshA* and *phoPQ* appear to contribute to β-lactam resistance by regulating membrane integrity. Notably, the overexpression of the uncharacterized membrane-associated protein, *ypfN*, has been shown to significantly enhance β-lactam resistance. We applied the genes identified from the screening into *Salmonella* Typhimurium and *Pseudomonas aeruginosa* strains, both critical human pathogens with antibiotic resistance, and observed their significant impact on β-lactam resistance. Therefore, these genes can potentially be utilized as therapeutic targets to control the survival of β-lactamase-producing bacteria.

## Introduction

β-lactam antibiotics including ampicillin, penicillins, cephalosporins and carbapenems are widely used [1]. β-lactam antibiotics function by disrupting the cross-linking of peptidoglycan during cell wall synthesis [2]. They achieve this by directly binding to penicillin-binding proteins (PBPs), ultimately resulting in bacterial death [3]. This mechanism is effective because PBPs are vital proteins for peptidoglycan construction, which, in turn, plays a crucial role in the durability of the bacterial cell wall [4]. As a consequence of β-lactam treatment, the cell wall becomes susceptible to external damage, such as osmotic shock [5]. Unfortunately, the number of bacteria resistant to β-lactam antibiotics is increasing at a high rate every year and bacterial resistance to β-lactam antibiotics is becoming a very significant threat to public health.

Gram-negative bacteria commonly become resistant to β-lactam antibiotics through the acquisition of genes encoding β-lactamases [6]. β-lactamases enable bacteria to survive in the presence of β-lactam antibiotics by hydrolyzing the β-lactam amide bond [7]. The genes encoding β-lactamases can readily be transferred through horizontal gene transfer, so the prevalence of bacteria producing these β-lactamase has been steadily increasing worldwide [8]. Since the transmissible plasmids that encode β-lactamases often also encode genes resistant to other classes of antibiotics, β-lactamase-producing bacteria are difficult to treat, increasing the risk of morbidity and mortality [9,10]. The bacteria producing β-lactamase not only protect themselves from β-lactam antibiotics but also safeguard nearby bacteria without resistance to β-lactam antibiotics by hydrolyzing the β-lactam antibiotics in their environment [11]. Therefore, the detection and control of β-lactamase producing bacteria is crucial for the effective management of infectious diseases.

*Escherichia coli* is a genetically well-established organism and is one of the species that have acquired β-lactam resistance. It has long been known that mutations in the PBPs can affect *E. coli*’s very low basal level of resistance to β-lactam antibiotics [12,13]. Additionally, *E. coli* constitutively express chromosomally encoded β-lactamase AmpC at a very low level, though it is not inducible due to the absence of the transcriptional activator AmpR [14,15]. In contrast, *Pseudomonas aeruginosa*, which exhibit intrinsic resistance to a broad range of antibiotics, including β-lactams, the expression of the chromosomally encoded AmpC-β-lactamase is highly active and plays a significant role in β-lactam resistance [16]. While these intrinsic mechanisms contribute to baseline resistance, the acquisition of plasmid-borne β-lactamase genes, such as TEM-1 β-lactamase have further enhanced resistance in *E. coli*.

In this study, we employed *E. coli* harbouring a plasmid encoding TEM-1 β-lactamase for genetic screening due to its high prevalence among plasmid-encoded β-lactamases and its clinical significance [17]. TEM-1 β-lactamase is not only one of the most frequently observed β-lactamases in Gram-negative bacteria, but it also represents the progenitor of an extensive family of extended-spectrum β-lactamases (ESBLs), which have evolved through mutations that broaden their substrate specificity [8].

To systematically identify host factors that influence β-lactam resistance in the presence of plasmid-borne β-lactamases, we performed a genome-wide transposon sequencing (Tn-Seq) screen in *E. coli* expressing TEM-1 β-lactamase. These experiments resulted in the re-identification of genes previously known to be associated with β-lactam resistance, including genes encoding outer membrane-associated porins. We were also able to discover new genetic loci, *gshA*, *phoP* and *ypfN*, that are involved in β-lactam resistance that is dependent on β-lactamase expression. Notably, these genes can also influence the β-lactam resistance of *Salmonella* Typhimurium, another member of the Enterobacteriaceae family and intrinsically β-lactam-resistant *P. aeruginosa* isolates recovered from clinical and environmental sources.

## Materials and methods

### Bacterial strains, growth conditions and plasmid constructions

All *E. coli*, *S.* Typhimurium and *P. aeruginosa* strains, plasmids and primers used in this study are listed in supplementary Table 1. Growth conditions and construction of mutants and plasmids are described in supplementary information.

### Transposon-sequencing (Tn-Seq)

Transposon mutagenesis was conducted following the procedure outlined by Lai et al [17]. Generation of transposon library, selection condition, DNA preparation for sequencing and sequencing analysis are described in supplementary information.

### Generation of in-frame deletion mutants

The genes identified through Tn-seq screening were validated by constructing deletion mutants. The deletion alleles, sourced from the systematic Keio knockout strain collection were introduced into the *E. coli* wild-type strain through P1 transduction as previously described [18,19], and detailed procedures are described in supplementary information.

### Spotting assay

*E. coli*, *S.* Typhimurium and *P. aeruginosa* strains were cultured at 37°C until the OD_600_ reached 1.0, after which they were subjected to spotting assay to access antibiotic resistance. Appropriate antibiotics for plasmid maintenance and inducers for cloned gene expression were added to the medium as described in supplementary information.

### Minimal inhibitory concentration (MIC) testing and MIC shift assay

To determine the MIC of antibiotics, we performed broth microdilution tests in LB medium with appropriate antibiotics. MIC shift assay was performed for 15 days in serial cultivation and MIC was measured. The detailed methods are described in supplementary information.

### Phylogenetic tree and protein structure

Protein sequences of YpfN (acc. No.: Q2EET2) were used as queries for searching YpfN homologues in NCBI databases through BLASTP, using standard parameters. Accession number of YpfN holomogous proteins and generation of phylogenetic tree are described in supplementary information. The protein structure of YpfN is acquired from AlphaFold Protein Structure Database (EMBL-EBI) as described in supplementary information.

### Colony morphology assay and biofilm quantification

To assess biofilm formation, bacteria were grown on Congo red agar plates for up to 10 days at 20°C and observed the colony morphology as described in supplementary information. Biofilm formation was quantified using a crystal violet assay where bacteria were grown in a 96-well plate at 20°C for 7 days as described in supplementary information.

### Membrane permeability assay using CPRG

Membrane permeability was assessed using chlorophenol red-β-D-galactopyranoside (CPRG), which changes color upon hydrolysis by intracellular β-galactosidase. Bacteria were grown to mid-log phase, treated with IPTG and then spotted onto LB plates containing CPRG as described in supplementary information.

### Swarming motility and pigment production

The swarming motility test was performed to assess the ability of bacterial cells to move collectively across a semi-solid surface, which contains 0.5% agar as described in supplementary information. To observe pyocyanin production, the bacteria were cultured in LB medium. After incubation at 37°C for 24–48 hours, the production of pyocyanin was detected as described in supplementary information.

### Genome-wide association analysis

We analysed 2910 *E. coli* clinical genomes from NCBI for ampicillin resistance and identified SNVs against the MG1655 reference genome using bioinformatics tools, followed by logistic regression analysis as described in supplementary information.

## Results

### Tn-Seq reveals β-lactam resistance associated genes in β-lactamase producing *E. coli*

**Figure 1.**
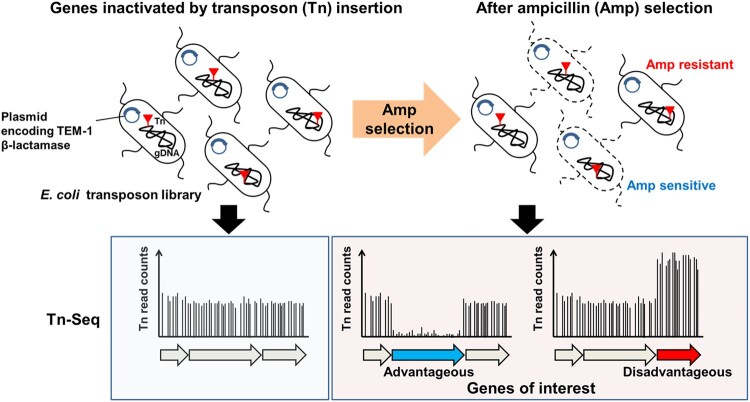
We generated a transposon (Tn) library in the *E. coli* K-12 strain containing the pBR322 plasmid encoding a β-lactamase. Ampicillin (Amp) resistance selections were then performed. Colonies resistant to ampicillin were pooled and subjected to next-generation sequencing. Depending on the transposon insertion site, alteration of transposon insertion frequency was observed in genes that are either advantageous or disadvantageous towards survival on media containing ampicillin.

Transposons insert into DNA with variable, in some cases nearly random, specificity. The genes disrupted by these insertions are frequently inactivated. Tn-seq technology systematically inserts transposons to create a mutant library containing mutations in every non-essential bacterial gene. By comparing the growth of the library under selective conditions with a control library, genes essential for bacterial growth under those conditions can be identified [20]. In this study, we sought to identify genes that affect antibiotic resistance in the MG1655 Δ*hsdR* strain of *E. coli* K-12 expressing the TEM-1 β-lactamase that is carried by the plasmid pBR322 (Figure 1 and Supplementary Table 1). We performed an ampicillin selection for the transposon mutant library. If the gene disrupted by the transposon insertion provided an advantage for growth in ampicillin, the frequency of transposon insertion would decrease during selection. If, on the other hand, the gene into which the transposon was inserted resulted in poorer growth in ampicillin, the frequency of transposon insertion would increase during selection.

Concentrations of ampicillin appropriate for performing the selection were obtained by conducting a spotting assay (Supplementary Figure 1). We report the results of a selection performed at an ampicillin concentration of 1.5 mg/ml, a concentration that allows the survival of only ∼10% of the plated cells. The cells from the transposon mutant library were collected before and after selection, and DNA was extracted and analysed by next-generation sequencing to determine the transposon insertion frequency (Materials and Methods). As a result, various genes related to ampicillin resistance were identified. In this study, β-lactam resistance refers to resistance relative to that of the parental (i.e. control) strain. Previously identified genes associated with β-lactam resistance, such as outer membrane proteins (OMPs) and the *marR* transcriptional regulator for multiple antibiotics, were rediscovered [21]. Additionally, several previously undiscovered genes related to β-lactam resistance were identified (see Supplementary Table 2), including proteases, polymerases and sigma factors, among others. These genes were confirmed by constructing in-frame deletion mutants followed by antibiotic resistance testing. Additionally, Tn-seq was repeatedly performed, which consistently identified the candidate genes (Supplementary Figure 2).

### Discovery of OMPs through Tn-Seq based screening

OMPs encode porins associated with β-lactam resistance, and as expected, they were identified in our study [22]. *E. coli* has various *omp* genes, including *ompF*, *ompA* and *ompC*. OmpF plays a major role in facilitating the passage of antibiotics, including β-lactams, through the outer membrane of bacterial cells [23]. Certainly, the transposon insertion frequency of the *ompF* gene notably increased following ampicillin selection in our study (Supplementary Figure 3). On the other hand, OmpA and OmpC are primarily involved in the stability of the outer membrane [23]. Notably, a significant decrease in transposon insertions were observed in *ompA* and *ompC*, indicating that the stability of the outer membrane via the OmpA and OmpC proteins is important for survival under β-lactam induced stress conditions (Supplementary Figure 3).

**Figure 2.**
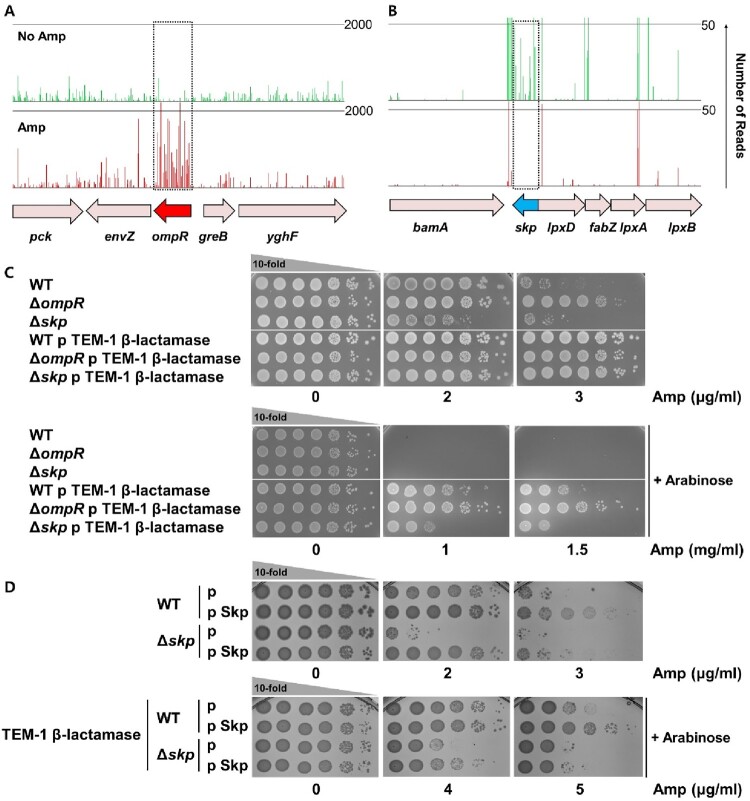
Transposon insertion frequency was increased 20-fold in the *ompR* gene, compared to the insertion frequency found without ampicillin selection (A). In contrast, the transposon insertion frequency was decreased 250-fold in the *skp* gene after ampicillin selection (B). Tn-Seq results before selection is shown in the upper panel as green peaks and that after selection is on the lower panel as red peaks. (C) The ampicillin resistance of the deletion mutants of *ompR* or *skp* was assessed through spotting assay. The pMB1-β-lactamase plasmid encodes TEM-1 β-lactamase under an arabinose-inducible promoter, and LB-agar plates containing arabinose were labelled as “+ arabinose”. (D) The deletion mutants were complemented by expressing the corresponding genes on the pCDFTrc plasmid.

OmpR is a transcriptional regulator that controls the expression of *omp* genes [24]. We found that the frequency of transposon insertion on the *ompR* gene was significantly increased in library pools grown in ampicillin relative to those grown on media lacking antibiotics. We generated an *ompR*-deficient mutant and verified that mutations in this gene are sufficient to increase resistance to the β-lactam ampicillin. One explanation for this result is that the inactivation of OmpR can increase β-lactam resistance by inhibiting the production of porins, such as OmpF, that allow β-lactams to penetrate the cell (Figure 2A) [25,26]. If this is true, then decreased permeability might affect the susceptibility to β-lactam antibiotics independently of the presence of a β-lactamase enzyme. We thus transformed a plasmid encoding β-lactamase under the control of an arabinose-inducible promoter to investigate whether β-lactam resistance depends on β-lactamase expression and, if so, whether it is related to the level of β-lactamase expression. We found that the *ompR* mutation significantly increases β-lactam resistance regardless of the presence or absence of β-lactamase expression (Figure 2C). In this genetic screening, we discovered these OMPs and their regulator, indicating the effectiveness of our experimental settings in discerning the distinct roles of OMPs in β-lactam resistance.

### Periplasmic chaperone Skp is involved in β-lactam resistance

It was observed that the frequency of transposon insertion of the *skp* gene significantly decreased after ampicillin selection (Figure 2B). The *skp* gene encodes a periplasmic chaperone that plays a role in the folding and stability of periplasmic and outer-membrane associated proteins, including OMPs [27]. Therefore, the change in β-lactam resistance observed in *skp* mutant may be attributed to alteration in its client proteins. Indeed, a *skp*-deficient mutant strain was created and proved to be sensitive to ampicillin, independent of the presence or absence of a β-lactamase (Figure 2C), suggesting that Skp-specific client protein(s) may be involved in β-lactam resistance, potentially affecting β-lactam permeability rather than directly influencing the folding of β-lactamase. Consistently, overexpression of *skp* fully restored β-lactam resistance in the *skp* deletion strain and increased β-lactam resistance, irrespective of the presence β-lactamase, in both wild-type and *skp* deletion background (Figure 2D). These findings strongly support the involvement of Skp in β-lactam resistance.

### Defect in glutathione synthesis pathway increases β-lactam resistance

**Figure 3.**
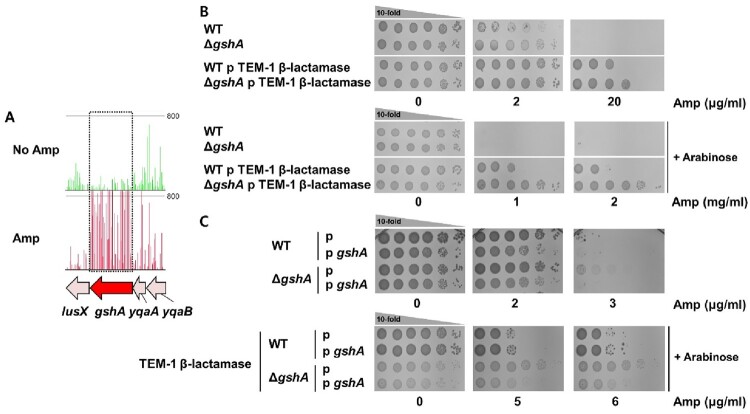
(A) The transposon insertion frequency was increased 23-fold in the *gshA* gene after ampicillin selection. (B) The ampicillin resistance of the *gshA* deletion mutant, both with and without TEM-1 β-lactamase, was evaluated through a spotting assay. (C) A complementation assay of *gshA* was conducted, both with and without TEM-1 β-lactamase.

We found that the frequency of transposon insertion in the *gshA* and *gshB* genes, which encode enzymes involved in the synthesis of glutathione (GSH), were significantly increased after selection and the independently constructed *gshA* mutants increase the resistance to ampicillin (Figure 3A,B, Supplementary Figure 3). Of note, in the presence of β-lactamase-encoded plasmids, the ampicillin resistance of the *gshA* mutant strain was increased 10-fold compared wild type strain background without addition of arabinose. However, the addition of arabinose, which induces expression of β-lactamase, significantly enhanced ampicillin resistance from 100 – to 1000-fold in the *gshA* mutant strain compared to wild type. This underscores the pivotal role of GSH synthesis in β-lactam resistance under conditions involving β-lactamase expression. The deletion phenotype can be restored by the overexpressing GshA, and wild-type strain exhibited decreased resistance to β-lactam (Figure 3C). Additionally, we conducted Minimum Inhibitory Concentration (MIC) tests and growth curve measurements, consistently confirming the β-lactamase-dependent ampicillin resistance phenotype of *gshA* mutant (Table 1, Supplementary Figure 5). While the MIC values of wild type and *gshA* mutant for ampicillin were the same, the MIC value of *gshA* mutant increased more than two-fold upon TEM-1 β-lactamase expression, demonstrating its β-lactamase-dependent ampicillin resistance. In addition, the spotting assay appears to be more sensitive than the MIC test in detecting marginal differences.

**Table 1.** Minimal inhibitory concentrations (MICs) of ampicillin for *E. coli* Δ*gshA*, Δ*ypfN* and Δ*phoP* mutants^a^.

Condition	Wild-type	Δ*gshA*	Δ*ypfN*	Δ*phoP*
TEM-1 β-lactamase not expressed^b^ Ampicillin (μg/ml)	9	9	8	6
TEM-1 β-lactamase expressed^c^ Ampicillin (mg/ml)	4	9	4	2

^a^ Mean MIC values were obtained from three biological replicates for each strain.

^b^ Strains do not possess the TEM-1 β-lactamase-containing plasmids.

^c^ Strains harbour the pMB1-β-lactamase plasmid, with expression induced by 1% arabinose.

We performed the MIC shift assay over 15 consecutive passages to assess the stability of antibiotic resistance in bacterial strain (Supplementary Figure 4). The first and fifth passages exhibited no significant differences in β-lactam resistance compared to the initial strain. However, by the tenth and fifteenth passages, the strain had decreased β-lactam resistance. This observation suggests that the maintenance of antibiotic resistance may be unstable over successive passage. Since we have noticed that *gshA* deletion leads to growth retardation even in the absence of ampicillin, suggesting that *gshA* serves a vital role in cellular physiology. Therefore, some spontaneous mutations may arise to compensate for this defect, which could in turn affect antibiotic susceptibility.

These findings strongly imply that GSH synthesis plays a role in β-lactam resistance. Glutathione plays a crucial role as an antioxidant in protecting cells from reactive oxygen species [28]. There are many genes whose level of expression is regulated by changes in glutathione levels and some protein’s functions are affected at a post translational level. These direct or indirect effects are likely involved in the mechanism by which mutating the glutathione genes affects β-lactam resistance.

### PhoPQ two component system is involved in β-lactamase-dependent β-lactam resistance.

**Figure 4.**
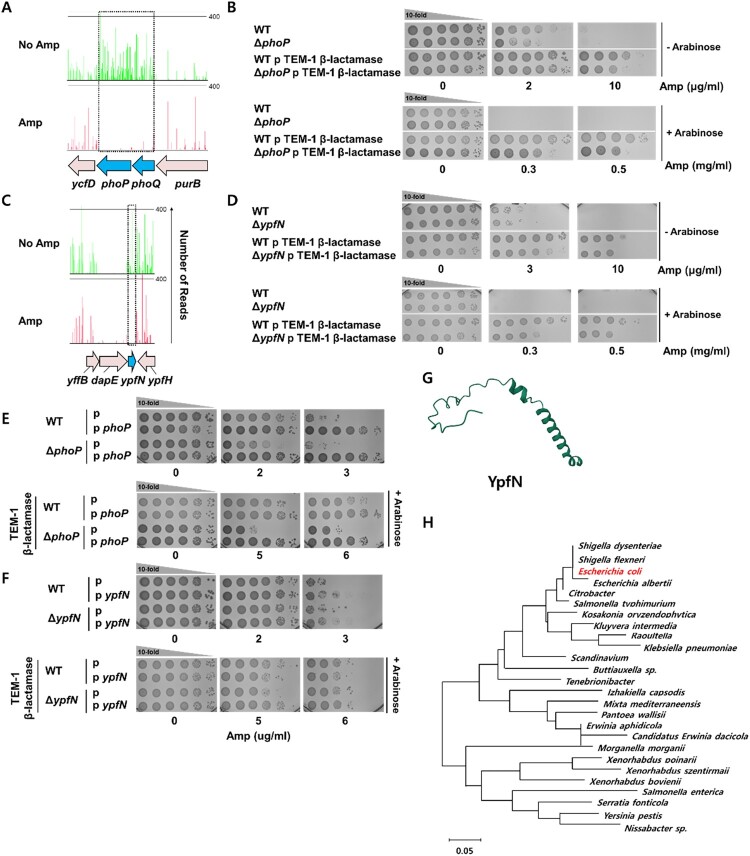
(A, C) The transposon insertion frequency decreased by 66-fold, 333-fold, and 333-fold in the *phoP*, *phoQ* and *ypfN* genes, respectively, compared to the insertion frequency found without ampicillin selection. (B, D) Ampicillin resistance of *phoP* and *ypfN* deletion mutants, with and without co-expression of TEM-1 β-lactamase, was examined by a spotting assay. (E, F) Complementation assays of *phoP* and *ypfN* was performed, both with and without TEM-1 β-lactamase. (G) The protein structure of YpfN predicted by Alphafold is displayed (AlphaFold Protein Structure Database, accession number: Q2EET2). (H) Phylogenetic analysis of YpfN protein homologues from different taxonomic groups of bacteria is presented.

We observed a significant decrease in the insertion of transposons into the *phoPQ* genes after ampicillin selection (Figure 4A). We created *phoP* mutant strains and confirmed their resistance to ampicillin. PhoPQ is a two-component system that responds to environmental signals such as low Mg^2+^ or low pH [29]. PhoQ is a membrane-bound sensor kinase that can detect environmental signals and phosphorylates the response regulator, PhoP [29]. The phosphorylated PhoP then binds to specific DNA sequences to regulate the expression of various genes involved in cell processes such as toxicity, adaptation to low Mg^2+^ environments, and acid resistance [29]. The *phoP* mutant exhibited moderate ampicillin sensitivity compared to the wild-type strain, and the *phoP* mutant strains expressing a β-lactamase showed increased ampicillin sensitivity (Figure 4B,E). Overexpression of PhoP increased β-lactam resistance in both wild-type and *phoP* deletion backgrounds, regardless of the presence of β-lactamase expression (Figure 4E). We observed similar results in the MIC test, where deletion of *phoP* gene moderately enhanced β-lactam susceptibility in a β-lactam dependent manner (Table 1). These findings suggest that PhoP is involved in β-lactam resistance, and the intracellular expression level of PhoP may be crucial for exerting β-lactamase-dependent β-lactam resistance. The regulons of PhoPQs can be involved in β-lactam resistance. It has been noticed that PhoPQ system regulates transcription of numerous genes and is involved in various pathways, such as the ATP-binding cassette transporter system, virulence, motility, biofilm formation and more [30]. Recently, it has been shown that PhoPQ can enhance structural integrity of the outer membrane by inducing modification of lipopolysaccharide in *E. coli*, resulting in increased antibiotic resistance [31]. Thus, we speculated that the stability of the outer membrane is one of the crucial factors contributing the high-level of β-lactam resistance in β-lactamase-producing strains. Indeed, we observed changes in membrane permeability after mutating *phoP* gene, as described in the section below.

### Uncharacterized inner-membrane associated protein YpfN is associated with β-lactam resistance

We found a significant decrease in the frequency of transposon insertion into the poorly characterized gene YpfN gene after ampicillin selection, implying that *ypfN* is involved in β-lactam resistance (Figure 4C). Deletion of this gene did not alter the resistance of strains to ampicillin relative to the wild type strain in the absence of β-lactamase-harbouring plasmids (Figure 4D). However, in the presence of β-lactamase-harbouring plasmids, *ypfN* deletions leads a 10∼100-fold increased susceptibility to ampicillin relative to the wild-type strain when the expression of the β-lactamase is induced. Furthermore, the overexpression of YpfN can significantly increase β-lactam resistance in both wild-type and *ypfN* deletion backgrounds in the absence of β-lactamase, indicating the involvement of *ypfN* in β-lactam resistance (Figure 4F). Additionally, *ypfN* can complement β-lactam resistance in the *ypfN* deletion background when β-lactamase is expressed. We also performed MIC tests on the *ypfN* deletion mutant with ampicillin. While only a slight decrease in ampicillin resistance was observed in the absence of β-lactamase, this is likely because the MIC test has limited sensitivity in detecting the moderate effect of *ypfN* on antibiotic resistance compared to the spotting assay, as mentioned above (Table 1).

YpfN is a small protein with 66 amino acids predicted, to be a mixture of α-helixes and random coils (Figure 4G), and is predicted to be associated with the inner membrane. YpfN is found not only in *E. coli* but also in various Gram-negative bacteria such as *Salmonella* and *Shigella* (Figure 4H, Supplementary Figure 6). Notably, YpfN is predominantly found in Gammaproteobacteria, as indicated by the UniProt database, where it appears in 98% (580 out of 600) of entries. Within Gammaproteobacteria, 96% (559 out of 580) of these are found in Enterobacterales. Interestingly, YpfN is strictly conserved in *E. coli*. To verify the presence of *ypfN*, we conducted a bioinformatic analysis using a dataset of 2675 and 3357 genomes of *E. coli* isolates that are resistant or susceptible to ampicillin, respectively, collected from the NCBI Pathogen Detection database. Surprisingly, 100% of *E. coli* genomes possess *ypfN*.

### *GshA* and *phoP* are involved in maintaining membrane integrity

**Figure 5.**
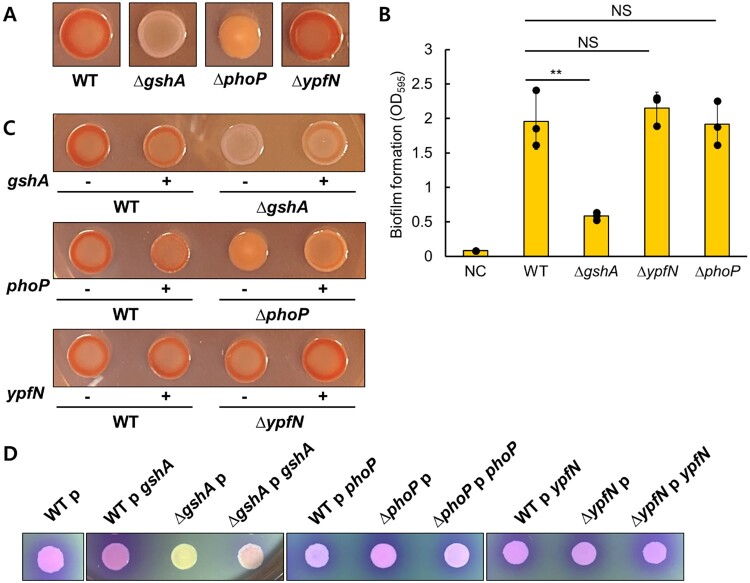
(A) Colony morphology was observed in the *gshA*, *phoP* and *ypfN* mutant strains on Congo red-containing plates. (B) Biofilm formation was quantified by crystal violet staining for each strain. The genes were overexpressed both in the wild-type background and in gene-deletion strains lacking the respective genes. The resulting phenotypes were assessed based on colony morphology on Congo red-containing plates (C) and membrane permeability on CPRG-containing plates (D).

Since biofilm formation and membrane permeability are directly linked to antibiotic resistance in bacteria, we examined these phenotypes. Notably, among the *gshA*, *phoP* and *ypfN* mutants, the *gshA* mutant exhibited a significantly reduced level of biofilm formation, as evidenced by both the colony color on Congo red-containing plates and crystal violet staining, which bind to biofilm matrix such as proteins and polysaccharides (Figure 5A,B). Congo red dye stains both cellulose and curli, which are major components of biofilm in *E. coli*, and a reddish color indicates a higher presence of biofilm-forming matrix [32]. The *phoP* mutant also exhibited a slightly less reddish color compared to the wild type. Overexpression of *gshA* and *phoP* was able to complement the phenotype on Congo red plates, while overexpression of *ypfN* did not significantly affect biofilm formation (Figure 5C). These results indicate that *gshA* and *phoP* are involved in biofilm formation in *E. coli*.

We also assessed membrane permeability using the CPRG dye, which reflects the integrity of cellular membranes. A purple color indicates membrane disturbance due to leakage of cytosolic content. Among the mutants, the *gshA* deletion mutant showed a whitish color, suggesting a significant enhancement in membrane integrity (Figure 5D). The *phoP* mutants exhibited a slightly lighter purple color compared to the wild type. Additionally, overexpression of *gshA* or *phoP* was able to complement the deletion phenotypes. These results suggest that *gshA* and *phoP* may contribute to antibiotic resistance by regulating membrane integrity. However, the *ypfN* mutant did not exhibit any notable changes in membrane integrity.

This result suggests that the change in membrane permeability may be associated with antibiotic resistance beyond β-lactams, particularly in the case of the *gshA* mutant. Therefore, we tested various antibiotics, including the aminoglycoside (kanamycin), tetracycline, and chloramphenicol (Supplementary Figure 7). The *gshA* mutant exhibited increased resistance to kanamycin and chloramphenicol, but not to tetracycline, indicating that the significant alteration of membrane integrity caused by *gshA* deletion may contribute to resistance not only to β-lactams but also to other antibiotics. In contrast, the *phoP* and *ypfN* genes did not affect resistance to other antibiotics.

### *GshA*, *phoP* and *ypfN* alter β-lactam resistance of *S.* Typhimurium and *P. aeruginosa*

**Figure 6.**
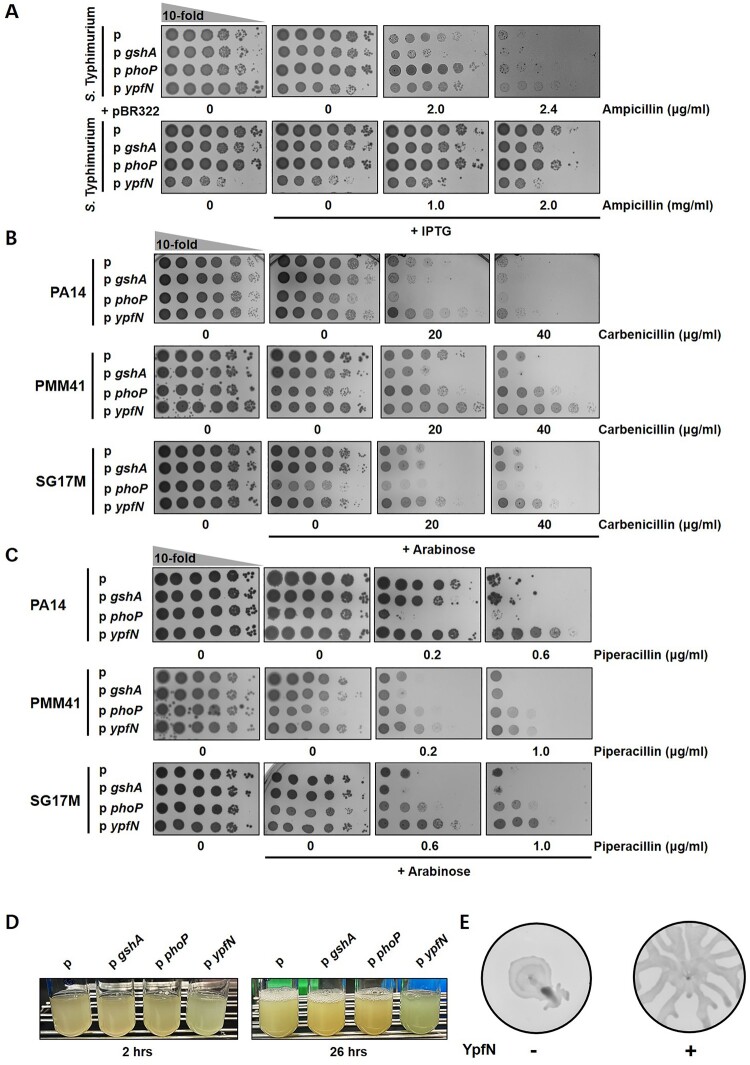
Assessment of β-lactam resistance and associated phenotypes in *S.* Typhimurium and *P. aeruginosa* isolates. (A) Spotting assays assessing β-lactam resistance in *S.* Typimurium on ampicillin-supplementeed media under conditions without (top) and with (bottom) β-lactamase expression. (B and C) Spotting assays evaluating β-lactam resistance in *P. aeruginosa* strains on media containing carbenicillin (B) and piperacillin (C). The strains tested include PA14 (reference strain, wound isolate), PMM41 (clinical isolate), and SG17M (environmental isolate). Pyocyanin production (D) and swarming motility (E) were monitored in the *ypfN*-expressing *P. aeruginosa* PMM41 strain.

Building upon our identification of ampicillin resistance-associated genes in *E. coli*, we extended our investigation to *S.* Typhimurium, another clinically significant pathogen within the Enterobacteriaceae family, to assess the universality of these resistance mechanisms across related species. We overexpressed the *E. coli* genes *gshA*, *phoP* and *ypfN* in *S.* Typhimurium using the pCDFTrc plasmid system. In addition, to evaluate the impact of β-lactamase expression, we co-transformed the strains with the pBR322 plasmid, which constitutively express the TEM-1 β-lactamase. Our results indicated that overexpression of *gshA*, *phoP* and *ypfN* in *S.* Typhimurium altered ampicillin resistance, mirroring the phenotypes observed in *E. coli* (Figure 6A). In the context of co-expression with pBR322, only *gshA* overexpression led to increased susceptibility to β-lactam antibiotics, while overexpression of the other genes did not significantly alter resistance levels compared to the wild-type strain. These findings suggest that the roles of *gshA*, *phoP* and *ypfN* in ampicillin resistance are conserved within Enterobacteriaceae, at least in *E. coli* and *S.* Typhimurium. However, in the presence of TEM-1 β-lactamase, *gshA* overexpression specifically affects β-lactam susceptibility, indicating species-specific differences.

*P. aeruginosa* is an opportunistic human pathogen with intrinsic resistance to various antibiotics, including β-lactams [33]. In particular, multidrug-resistant (MDR) *P. aeruginosa* poses a significant public health threat. Unlike *E. coli*, *P. aeruginosa* actively expresses a chromosomally encoded AmpC β-lactamase [34]. Therefore, we applied the identified genes in *P. aeruginosa*. Three *P. aeruginosa* strains were used in this study: the reference strain PA14, the clinical isolate PMM41, and the environmental isolate SG17M [35-39]. The genes *gshA*, *phoP* and *ypfN*, originated from *E. coli*, were overexpressed using the broad-host-range plasmid pJN105, which contains an arabinose-inducible promoter [40]. In *P. aeruginosa*, carbenicillin and piperacillin are often preferred over ampicillin due to its broader spectrum and reduced susceptibility to β-lactamase degradation [41]. Therefore, we used carbenicillin and piperacillin to assess the effects of gene overexpression in *P. aeruginosa*.

Consistent with the results in *E. coli*, *gshA* expression reduced resistance to carbenicillin and piperacillin in *P. aeruginosa* PA14 and PMM41strains (Figure 6B,C). However, overexpression of *phoP* caused increased sensitivity to carbenicillin and piperacillin in PA14, an opposite phenotype compared to *E. coli*. We hypothesize that this difference may be due to the variation in PhoP specificity between species, as the *P. aeruginosa* PhoP protein shares only 52% amino acid sequence identity with that of *E. coli*. To test this, we expressed the *P. aeruginosa* version of *phoP* from the pJN105 plasmid and found that its overexpression increased resistance to carbenicillin and piperacillin (Supplementary Figure 8).

Unlike *phoP* and *gshA*, *ypfN* does not have a homologous protein encoded in the core genome of *P. aeruginosa*. Notably, overexpression of *ypfN* increases high resistance to carbenicillin and piperacillin in all *P. aeruginosa* strains, consistent with results observed in *E. coli* but with a much stronger effect (Figure 6B,C). Furthermore, *ypfN* was found to enhance virulence-associated phenotypes, including increased pyocyanin production and motility. These findings suggest that *ypfN* may serve as a positive factor contributing to the pathogenic potential of *P. aeruginosa* (Figure 6D,E).

Although *ypfN* is not encoded in the core genome of *P. aeruginosa*, it has been found in certain isolated *P. aeruginosa* strains, such as the clinical isolate PA-K152. The identical *ypfN* gene sequence was also found in clinical isolates of *E. coli*, such as the p1-49 strain. Based on GC-content analysis, *ypfN* in *P. aeruginosa* appears to be part of a genomic island (Supplementary Figure 9), suggesting that horizontal gene transfer may have introduced *ypfN* into *P. aeruginosa*, potentially contributing to increased β-lactam resistance.

### Association with ampicillin susceptibility through genome-wide association analysis

We used publicly available *E. coli* genomes from clinical isolates with associated ampicillin susceptibility phenotype data (*n* = 2910; susceptible isolates 43.7%, resistant or intermediate isolates 56.3%), and tested if the mutations in the coding regions or promoters of these Tn-Seq-discovered genes are associated with ampicillin phenotypes among clinical isolates. Firstly, we found that frequency of polymorphic sites in these genes was not above the genome-wide trend. Indeed, we did not find polymorphic sites (major allele frequency < 99%) in the CDS of *skp* and *ypfN*, neither in the promoter regions of any of the five genes; *ompR*, *skp*, *gshA*, *phoP* and *ypfN*. We found single polymorphic site per each of *gshA*, *ompR* and *phoP* (Supplementary Figure 11). Genome-wide association analysis between the genome-wide SNPs (*n* = 7268) and ampicillin phenotypes indicated that variants in *gshA* and *ompR* were not strongly associated with the categorical resistance phenotype (*gshA*, FDR = 0.042, rank = 5391 among the genome-wide variants; *ompR*, FDR = 0.97, rank = 7228), and a variant in *phoP* had a slightly stronger association (FDR = 1.7E-13, rank = 1131).

## Discussion

**Figure 7.**
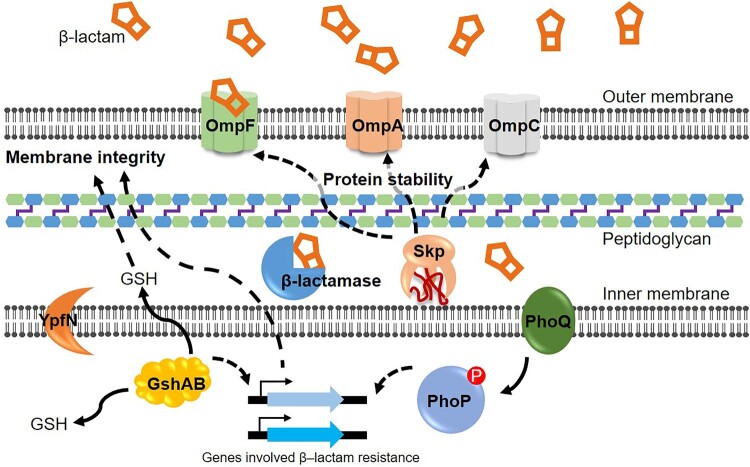
Various proteins plays distinct roles in β-lactam antibiotics resistance. OMPs, such as OmpF, OmpA and OmpC, contribute to β-lactam resistance by regulating permeation of antibiotics into cells and maintaining membrane stability. Skp, a periplasmic chaperone, is responsible for the stable insertion of OMPs into the outer membrane and may potentially have unidentified client proteins associated with β-lactam resistance. GshA and GshB are involved in synthesizing GSH, present in the periplasmic and cytoplamic spaces. The GSH levels influence the expression of various genes potentially involved in β-lactam resistance and may impact membrane integrity. PhoPQ, a two-component system, has regulons that may contribute to β-lactam resistance, presumably through regulating membrane integrity. YpfN is a protein with an unknown role predicted to be located in the inner membrane.

In this study, we have identified various genes involved in β-lactam resistance using the Tn-Seq method. Given that the acquisition of β-lactamase-encoding genes through horizontal gene transfer is a major contributor to β-lactam resistance, we conducted the screening in a background of β-lactamase-producing *E. coli* strain. Consequently, we discovered several previously unknown genes associated with β-lactam resistance (Figure 7). Significantly, some of these genes exhibited a β-lactam-resistant phenotype dependent on the expression of β-lactamase.

Our experimental findings suggest that Skp is implicated in β-lactam resistance through its client proteins, rather than being directly involved in the folding or stability of β-lactamase. In *E. coli*, Skp plays a crucial role as a chaperone in maintaining periplasmic protein homeostasis [42]. The loss of *skp* expression increases resistance to antimicrobial peptides through the modulation of OMPs [43]. Although this case is contrasts with what we observed in β-lactam resistance, these results suggest that Skp plays a crucial role in various antimicrobial compounds, presumably through the modulation of its client proteins.

It has been proposed that some antibiotics including β-lactams, stimulate the production of reactive oxygen species (ROS), contributing to bactericidal activity [44,45]. However, the role of ROS-mediated killing of bacteria is a topic of debate among research groups [46,47]. Notably, various genes involved in the response to ROS, such as *gor*, *trxABC*, *grxABCD*, *katGE*, *sodABC*, did not show altered transposon insertion frequencies (Supplementary Figure 10). Exceptionally, the *gshA* and *gshB* genes, encoding enzymes for synthesizing GSH, an important redox factor with antioxidant activity, did show altered transposon insertion frequencies. Increased β-lactam resistance was confirmed in the *gshA* mutant, suggesting that involvement of GSH in β-lactam resistance is not through its redox properties but potentially through other mechanisms, such as changes in gene expression patterns or regulating membrane integrity.

The PhoPQ two-component system regulates various genes, and genes with altered expression might be involved in β-lactam resistance. A recent study characterized the involvement of PhoPQ is the β-lactam antibiotic meropenem in *Enterobacter cloacae* [31]. PhoPQ expression enhances the structural integrity of the outer membrane through the modification of lipopolysaccharide (LPS) [31]. The *arn* operon, which encodes enzymes responsible for synthesizing and transferring positively charged L-Ara4N moieties to lipids, is involved in LPS modification and is upregulated by PhoPQ [31]. In *E. coli*, the *arn* operon is conserved and regulated by the BaeRS two-component system [31]. However, it is unknown whether the *arn* operon is regulated by the PhoPQ system. Additionally, in our experimental results, the alteration of transposon insertion frequency was not observed (Supplementary Figure 8), suggesting the involvement of other genetic resistance mechanisms. Therefore, it is likely that *E. coli* has other genetic factors involved in *PhoPQ*-mediated β-lactam resistance, which need further verification.

In this study, we first unveiled the role of YpfN in β-lactam resistance. YpfN is predicted to be an inner membrane-associated protein and is present in various Gram-negative bacteria. Notably, *ypfN* is prevalent among Gammaproteobacteria, particularly in Enterobacterales. YpfN is highly conserved within the core genome of *E. coli*, suggesting that, despite its unknown function, *ypfN* is evolutionarily preserved and likely plays a crucial role in *E. coli*.

In the MIC test with serial passaging, bacterial strains can lead to significant changes in antibiotic resistance profiles. In this study, after 15 consecutive passages, strains that initially exhibited high ampicillin resistance showed a reduction to wild type susceptibility levels, while those with low initial resistance developed increased resistance comparable to the wild type (Supplementary Figure 4). These observations suggest that genetic mutations, metabolic costs associated with resistance, and selective pressures during passaging may influence the stability of antibiotic resistance. Such findings underscore the dynamic nature of a β-lactam resistance and highlight the importance of monitoring resistance patterns over time.

*P. aeruginosa* is increasingly recognized for its intrinsic resistance to various antibiotics, including β-lactams, primarily due to the expression of chromosomally encoded AmpC β-lactamase. In this study, we identified that the genes *gshA*, *phoP* and *ypfN* also play a role in mediating β-lactam resistance in *P. aeruginosa*. As *P. aeruginosa* harbours homologous genes for *gshA* and *phoP*, these genes represent potential targets for modulating antibiotic resistance in this pathogen. While *P. aeruginosa* does not typically possess the *ypfN* gene in its genome, genomic database searches have identified certain *P. aeruginosa* isolates that carry *ypfN* within a genomic island, likely acquired through horizontal gene transfer. This finding may indicate that *ypfN* could be an emerging genetic factor contributing to β-lactam resistance in *P. aeruginosa*. Additionally, *ypfN* appears to influence various physiological processes in *P. aeruginosa*, including pyocyanin production and swarming motility.

Through this Tn-Seq-based genetic screening, we identified several genes involved in β-lactam resistance. The further study of the molecular mechanisms of action of these genes is required as this may allow then to become potential targets for the development of antibiotics of β-lactamase producing bacteria.

## Supplementary Material

Supple_AmpRS_rev2_CL37.docx

supplementary table 3.xlsx

## Data Availability

Data will be made available on request.
