# Supplementary material for: Identification of host genetic factors modulating β-lactam resistance in Escherichia coli harbouring plasmid-borne β-lactamase through transposon-sequencing
Source: Emerg Microbes Infect. 2025 Apr 15;14(1):2493921. doi: 10.1080/22221751.2025.2493921 (PMC12024506; doi:10.1080/22221751.2025.2493921)
Supplement: Supple_AmpRS_rev2_CL37.docx [file TEMI_A_2493921_SM7955.docx]

**SUPPLEMENTARY INFORMATION**

**Identification of Host Genetic Factors Modulating β-Lactam Resistance in *Escherichia coli* harboring plasmid-borne β-lactamase through Transposon-Sequencing**

**AUTHORS AND AFFILIATIONS:**

Hyunhee Kim^1,2^, Travis Bell^3^, Kihyun Lee^5^, Jeongyun Jeong^1^, James CA Bardwell^3,4^, Changhan Lee^1*^

^1^ Department of Biological Sciences, Ajou University, Suwon, South Korea

^2^ Research Institute of Basic Sciences, Ajou University, Suwon, South Korea

^3^ Department of Molecular, Cellular, and Developmental Biology, University of Michigan, Ann Arbor, Michigan, USA

^4^ Howard Hughes Medical Institute, University of Michigan, Ann Arbor, Michigan, USA

^5^ CJ Bioscience

* Correspondence:

Changhan Lee

leec@ajou.ac.kr

**Supplementary materials and methods**

**Bacterial strains, growth conditions, and plasmid constructions**

The strains used in this study are listed in Supplementary Table 1. All *E. coli* strains used in this study are derivatives of *E. coli* K-12. The strain MG1655 Δ*hsdR* was used as wild type, exhibiting enhanced transformation efficiency due to the absence of the type I restriction enzyme EcoKI endonuclease. The in-frame deletion mutant construction is described as below. The cells were grown at 37°C in Luria–Bertani (LB) broth supplemented with appropriate antibiotics (25 μg/ml kanamycin and 17 μg/ml tetracycline). Genes identified through screening were cloned into pCDFTrc for in vivo complementation purposes (see Supplementary table 1 for details). *S.* Typhimurium and *P. aeruginosa* strains PA14, PMM41 (clinical isolates), and SG17M (environmental isolate) were cultured at 37°C in LB medium supplemented with appropriate antibiotics. (30 μg/ml gentamycin). pCDFTrc and pMB1 plasmids were used to overexpress the genes and TEM-1 β-lactamase. pJN105 plasmid was used to overexpress the genes in *P. aeruginosa*. Primer sequences and the corresponding restriction enzymes used for cloning are provided in supplementary table 1.

**Transposon-sequencing (Tn-Seq)**

Transposon mutagenesis was conducted following the procedure outlined by Lai et al [[1](#_ENREF_1)]. In summary, MG1655 Δ*hsdR* pBR322 strains were subjected to mutagenesis using the Ez-Tn5 <Kan-2> transposome from Epicentre. This transposome can integrate into random positions in the genome, carrying a kanamycin resistance gene with approximately one insertion per genome. Mutant strains with transposon insertions were obtained by selecting for kanamycin resistance on LB-Kanamycin agar plates at 37°C. A pool of colonies, ranging between 7 × 10^5^ and 10^6^, was collected from the LB-Kanamycin plates and suspended in LB broth. Glycerol was added to the suspension (OD_600_ = ~80) to achieve a final concentration of 16%. The resulting mutant library was stored at −80°C. Subsequently, 140 μl of the mutant library cell stock was inoculated into 50 ml of LB broth and cultured at 37°C with shaking until the OD_600_ reached ~1. The library cells were then plated on LB agar with ampicillin concentrations of 1.5 mg/ml and incubated at 37°C for 16 h. Colonies that developed on these LB-ampicillin plates were harvested by scraping and suspended in LB broth.

Sequencing libraries for Tn-seq were prepared following the method previously outlined by Lai et al [[1](#_ENREF_1)]. Genomic DNA was extracted from the collective mutant sample using the Wizard Genomic DNA Purification Kit (Promega) and subsequently fragmented using NEBNext dsDNA Fragmentase (NEB) at 37°C for 25 min. The fragmented DNA was then purified using Agencourt AMPure XP beads (Beckman Coulter, Inc.) and eluted in water. Following purification, the fragmented DNA underwent treatment with terminal deoxynucleotidyl transferase (TdT; Promega) in a reaction involving dCTP and ddCTP to append poly-dC at the 3′ end of the DNA, conducted at 37°C for 1 h. Subsequently, TdT was deactivated at 75°C for 20 min. The TdT-treated DNA was purified using a DTR gel filtration cartridge (EdgeBio).

The DNA treated with TdT served as a template for PCR amplification of transposon junctions, utilizing the Easy-A HiFi Cloning System from Agilent Technologies with the primers PolyG-1st-1 and Tn5-1st-1 (Supplementary Table 1). Subsequently, a second nested PCR was performed to amplify the transposon junctions and incorporate the sequencing barcode, using NEBNext Multiplex Oligos for Illumina (NEB) with Tn5-2nd-1 as primers. The final PCR product underwent electrophoresis on a 2% agarose gel, and fragments falling within the 200 to 500 bp range were excised and purified using a QIAquick Gel Extraction Kit (Qiagen). The resulting purified libraries were then sequenced at the University of Michigan Sequencing Core on a HiSeq 2500 (Illumina) in a single-end run.

Sequencing reads, totaling over 12,000,000 reads per each sample, were normalized across the samples, and aligned to the *E. coli* MG1655 genome (NCBI NC_000913). The Bowtie mapping method was employed for the read mapping onto the genome, excluding transposon insertions in the pBR322 plasmid [[2](#_ENREF_2)]. The Sanger Artemis Genome Browser and ggplot2 R package version 3.5.0 were utilized for visualizing and locating transposon insertion sites. Fold change enrichment under ampicillin selection conditions compared to LB without ampicillin was calculated following the methodology outlined in Lai et al [[1](#_ENREF_1)]. Transposons were inserted throughout the entire genome, both before and after selection (Supplementary Fig. 1). A Quantile-Quantile (Q-Q) plot that compares the insertion density of individual genes before and after selection reveals a positive linear correlation between the two variables, indicating that an increase in the mean insert density without selection corresponds to an increase in the mean insert density with ampicillin selection. The data points are closely aligned with the trend line, demonstrating a strong linear without much deviation. For further validation, we selected genes that showed more than 10-fold change in transposon insertion frequency, with a Mann-Whitney U test p-value below 0.001. Genes already known to be associated with β-lactam resistance were excluded from the verification process. The resulting gene list underwent a spotting assay for final confirmation. To confirm the candidate gene’s association with ampicillin resistance, in-frame deletion mutants were constructed as described above, and their phenotype on ampicillin resistance was tested. Additionally, Tn-seq was repeatedly performed, resulting in reproducible data.

**Generation of in-frame deletion mutants**

The genes identified through Tn-seq screening were validated by constructing deletion mutants and subsequent testing their antibiotic resistance. The deletion alleles, sourced from the systematic Keio knockout strain collection were introduced into the *E. coli* wild-type strain through P1 transduction as previously described [[3](#_ENREF_3),[4](#_ENREF_4)]. To avoid overlap with antibiotic resistance marker on a plasmid, the single kanamycin insertion mutants underwent transformation with pCP20, a plasmid encoding a temperature-sensitive flippase. This plasmid was utilized to eliminate the kanamycin-resistant cassette from the chromosome, generating a kanamycin-sensitive deletion [[4](#_ENREF_4)].

**Spotting assay**

Colonies of the *E. coli*, *S*. Typhimurium and *P. aeruginosa* strains were picked from LB plates with appropriate antibiotics and then cultured overnight in LB broth containing the corresponding antibiotics with shaking at 37°C. The overnight cultures were diluted 100-fold in LB broth and incubated with shaking at 37°C until the OD_600_ reached 1.0. To investigate the impact of β-lactamase expression, pMB1 plasmid was utilized, wherein TEM-1 β-lactamase was cloned into pBR322. In complementation experiments, each candidate gene cloned into the pCDFTrc vector was induced with 0.2 mM IPTG and for pMB1 co-expression, 0.2% arabinose was added to induce TEM-1 β-lactamase, when the culture OD_600_ reached 0.3–0.4; incubation continued 1 hour with shaking. For *P. aeruginosa*, strains with overexpression of the genes, pJN105 plasmid was used and 1% of arabinose was added to induce each candidate genes at OD_600_ of 0.3-0.4 and incubated for 2 hours with shaking. The cells were then diluted 10-fold in the range of 10^−1^ to 10^−6^, and 3 μl was spotted onto LB plates containing different concentrations of antibiotics and supplemented with IPTG and arabinose as stated in the figure. The spotted plates were incubated at 37°C for 12–14 hours. For the experimental sets using two plasmids to express both β-lactamase and the identified genes, a relatively low concentration of ampicillin was used, likely due to co-expression issues. Such issues often arise even when using two compatible vectors with different origins of replication. The experiments were repeated at least twice, and the results were consistently reproduced.

**Minimal inhibitory concentration (MIC) and Growth curve testing**

To determine the MIC of antibiotics, we employed the broth microdilution method in LB medium supplemented with the appropriate antibiotics [[5](#_ENREF_5)]. An overnight culture of *E. coli* was diluted to an OD_600_ of 0.001 in fresh LB medium containing the appropriate concentration of ampicillin and 1% of arabinose, as required. Total of 200 μl of this prepared culture was dispensed into each well of a 96-well microtiter plate. The plate was incubated at 37°C for 18 to 22 hours. To better distinguish differences in antibiotic resistance among mutants, various antibiotic concentrations in a linear series (1, 2, 3, 4 µg/mL, etc.) were examined, rather than the conventional two-fold dilution series. After incubation, cell growth was assessed by measuring the OD_600_ value using a plate reader. No cell growth was determined if the OD_600_ values were comparable to those of the medium-only control wells. In addition, antibiotic resistance for each mutant was determined by comparing its growth to that of the wild-type strain at the same antibiotic concentrations. Following incubation, bacterial growth was assessed using a Tecan M flex plate reader for checking the cell growth. For growth curve analysis, cells were prepared similarly to the MIC assay. OD_600_ was measured every 10 minutes using a Tecan M flex plate reader over 14 hours. In the growth curve analysis, a mutant was considered to have increased susceptibility if it exhibited no growth or delayed growth compared to the wild-type strain at the same antibiotic concentration. MIC values and growth curves were determined as the mean of three biological replicates. This approach allowed for continuous monitoring of bacterial growth dynamics. The experiments were repeated at least twice, and the results were consistently reproduced.

**MIC shift assay**

To assess the stability of antibiotic resistance over time, we conducted a 15-day serial cultivation experiment. A single colony was inoculated into LB media for deletion strains and LB media with the addition of appropriate antibiotics and inducers for strains expressing TEM-1 β-lactamase. The culture was incubated at 37°C for 24 hours. Each day, a 1:100 dilution of the 24-hour culture was made into fresh LB medium containing the same antibiotics and inducers. This process was repeated daily for a total of 15 days. Aliquots from the cultures were collected and stored at -80°C on days 1, 5, 10, and 15. Samples from each time point were streaked onto LB agar plates, with and without the appropriate antibiotics, to isolate single colonies. Single colonies from these plates were inoculated into LB broth, with or without antibiotics as appropriate, and subjected to MIC testing as previously described [[6](#_ENREF_6)]. The experiments were repeated at least twice, and the results were consistently reproduced.

**Phylogenetic tree**

Protein sequences of YpfN (acc. No.: Q2EET2) were used as queries for searching YpfN homologs in NCBI databases through BLASTP, using standard parameters. The accession number of the YpfN homologous proteins are stated in the figure legend (Supplementary Fig. 4). These database searches retrieved YpfN homologs with an identity down to 58%. The alignment of proteins was conducted using Clustal Omega with standard parameters [[7](#_ENREF_7)]. Subsequently, the aligned sequences underwent phylogenetic analysis by applying neighbor-joining in MEGA11 [[8](#_ENREF_8)].

**Protein structure**

The protein structure of YpfN is acquired from AlphaFold Protein Structure Database (EMBL-EBI). The accession number is UPF0370 for YpfN protein. The protein structure was visualized by Mol* 3D Viewer provided by RCSB PDB, and the default values of parameters were used.

**Colony morphology assay on Congo red-containing medium**

To assess biofilm-related phenotype, colony morphology assay on Congo red-containing medium was performed [[9](#_ENREF_9)]. Bacterial strains were cultured overnight in Tryptone broth (10 g/L) without salt with appropriate antibiotics at 37°C with shaking. The following day, cell was diluted to 0.025 of OD_600_, and 10 µL of each diluted culture was spotted onto Congo red agar plates, which were prepared by supplementing TB broth without salt with 40 µg/mL Congo red, 20 µg/mL Coomassie Brilliant Blue G-250, appropriate antibiotics and 0.2 mM IPTG to induce a gene in the plasmid. The plates were incubated at 20°C until 10 days. Colonies were photographed and observed every day from the 3rd to the 10th day of culture. Strains with enhanced biofilm production or increased extracellular polysaccharide synthesis exhibited dark red or black colonies, whereas strains with reduced biofilm formation or matrix production appeared pink or white.

**Crystal Violet-based assay for quantification of biofilm formation**

To quantify biofilm formation, a crystal violet assay was performed as follows [[10](#_ENREF_10)]. Bacterial strains were grown overnight in LB broth with appropriate antibiotics at 37°C with shaking. The overnight cultures were then diluted to 0.01 of OD_600_ in fresh LB broth with appropriate antibiotics and 0.2 mM IPTG to induce transformed gene expression, and 200 µL of each diluted culture was inoculated into a sterile, flat-bottom 96-well polystyrene microtiter plate. The plate was incubated statically at 20°C for 7 days to allow biofilm formation. After incubation, the planktonic cells were gently removed by aspirating the medium, and each well was washed three times with 250 µL of sterile phosphate-buffered saline (PBS) to remove non-adherent cells. The biofilms were then stained with 250 µL of 0.4% crystal violet solution for 10 minutes at room temperature. Excess stain was removed by rinsing the wells three times with PBS. The plate was air-dried, and the bound crystal violet was solubilized by adding 250 µL of DMSO to each well. The absorbance of the solubilized crystal violet was measured at 595 nm using a microplate reader. Biofilm formation was quantified by comparing the absorbance values of each sample to the negative control (wells with sterile LB broth only). All experiments were performed in triplicate, and the data were presented as mean values with standard deviations. The experiments were repeated, and the results were consistently reproduced.

**Membrane permeability assay using CPRG**

To assess membrane permeability, we utilized the substrate chlorophenol red-β-D-galactopyranoside (CPRG), which changes color upon hydrolysis by intracellular β-galactosidase, indicating increased membrane permeability [[11](#_ENREF_11)]. Bacterial strains were cultured overnight in LB broth at 37°C with shaking. The following day, the cultures were diluted 1:100 in fresh LB broth and grown to mid-log phase (OD_600_ = 0.4-0.6). IPTG of 0.2 mM was added and incubate for 1 hour at 37°C with shaking. Cell was diluted to 0.5 of OD_600_ and serially diluted from 10^-1^ to 10^-6^ in 10-fold dilution. Aliquots of 2 µL were spotted onto LB plates with appropriate antibiotics, 0.2 mM IPTG and 20 ug/ml CPRG. After 12 ~ 24 h incubation at 37°C, photographs of the plates were taken and analysed. The experiments were repeated at least twice, and the results were consistently reproduced.

**Swarming motility**

The swarming motility test was performed to assess the ability of bacterial cells to move collectively across a semi-solid surface [[12](#_ENREF_12)]. A bacterial suspension was inoculated onto the center of a 0.5% agar plate and incubated at 37°C. Plates were monitored for the formation of a characteristic radial pattern, indicating swarming motility. The experiments were repeated at least twice, and the results were consistently reproduced.

**Pigment production**

To observe pyocyanin production in *P. aeruginosa*, the bacteria were cultured in LB medium with appropriate antibiotics [[13](#_ENREF_13)]. After incubation at 37°C for 24-48 hours, the production of pyocyanin was detected by the appearance of blue and green pigments. The intensity of the pigments was visually assessed and documented. The experiments were repeated at least twice, and the results were consistently reproduced.

**Genome-wide association analysis**

We collected *E. coli* genomes in NCBI Pathogen Detection database which have associated ampicillin phenotype data and categorized as clinical isolates (n = 2910; by phenotype: S, 1272; I, 5; R, 1633) (Supplementary table 3; *E. coli* clinical isolate genomes with ampicillin phenotype).

We profiled single-nucleotide variants in each of these clinical isolates against the chromosome sequence of MG1655 (U00096.3) using an in-house method. We aligned the contigs of each clinical isolate genome on to the reference MG1655 chromosome using Minimap2 with “-x asm10” preset and “-a” option to produce SAM files. From the SAM files we derived variants in VCF format using samtools and bcftools. Polymorphic loci with major allele frequency greater than 99% were excluded from the further analysis. In the association test, we transformed the allele (i.e., base) with the highest frequency at the given locus to 0 (i.e., major allele), and the other alleles to 1 (i.e., minor alleles), and used logistic regression implemented in ‘glm’ function of R base package.

**Supplementary tables**

**Supplementary table 1.** List of strains, plasmids and primers

| **Strain** | **Genotype** | **Source/reference** |
| --- | --- | --- |
| MG1655 Δ*hsdR* | a *E. coli* K-12 strain that is defective in degradation of foreign DNA to improve transposon-derived mutagenesis efficiency but otherwise wild type | [[14](#_ENREF_14)] |
| CL195 | SQ765 pBR322 | This study |
| CL513 | SQ765 Δ*ompR* | This study |
| CL685 | SQ765 Δ*skp* | This study |
| TB03 | SQ765 Δ*gshA*::Kan | This study |
| TB61 | SQ765 Δ*phoP*::Kan | This study |
| TB61 | SQ765 Δ*ypfN*::Kan | This study |
| eHK145 | SQ765 Δ*gshA* | This study |
| eHK148 | SQ765 Δ*phoP* | This study |
| eHK146 | SQ765 Δ*ypfN* | This study |
| pHK98 | *Salmonella enterica* serovar Typhimurium strain 14028s | [[15](#_ENREF_15)] |
| CL1453 | *Pseudomonas aeruginosa* PA14 | [[16](#_ENREF_16)] |
| CL1456 | *Pseudomonas aeruginosa* SG17M | [[17](#_ENREF_17),[18](#_ENREF_18)] |
| CL1571 | *Pseudomonas aeruginosa* PMM41 | [[19](#_ENREF_19),[20](#_ENREF_20)] |
| **Plasmid** | **Description** | **Source/reference** |
| pBR322 | Expression vector; pMB1 ori, ampicillin^R^, tetracycline^R^ | [[21](#_ENREF_21)] |
| pMB1-β-lactamase | Modified pBR322 vector; containing the *araC* gene and the *bla* gene under arabinose-inducible control | [[22](#_ENREF_22)] |
| pCDFTrc | Expression vector; CloDF13-derived CDF ori, Kanamycin^R^ | [[14](#_ENREF_14)] |
| pCDFTrc-*skp* | pCDFTrc containing *skp* gene | This study |
| pCDFTrc-*gshA* | pCDFTrc containing *gshA* gene | This study |
| pCDFTrc-*phoP* | pCDFTrc containing *phoP* gene | This study |
| pCDFTrc-*ypfN* | pCDFTrc containing *ypfN* gene | This study |
| pJN105 | A broad-host range vector with arabinose inducible *araBAD* promoter; pBBR1ori, Gentamycin^R^ | [[23](#_ENREF_23)] |
| pJN105-*gshA* | pJN105 containing *gshA* gene | This study |
| pJN105-*phoP* | pJN105 containing *phoP* gene | This study |
| pJN105-*ypfN* | pJN105 containing *ypfN* gene | This study |
| pJN105-*Pae PhoP* | pJN105 containing *phoP* gene derived from *P. aeruginosa* PA14 strain | This study |
| **Primer** | **Sequence** | **Purpose** |
| skp-pCDFTrc-F | 5’-GCGGGATCCGGGATGGTAAGGAGTTTATT-3’ | Cloning *skp* into pCDFTrc (Restriction site: BamHI-XhoI) |
| skp-pCDFTrc-R | 5’-GCGCTCGAGTTATTTAACCTGTTTCAGTACG-3’ | Cloning *skp* into pCDFTrc (Restriction site: BamHI-XhoI ) |
| gshA-pCDFTrc-F | 5’- GCGGGATCCTTGACAGGCGGGAGGTCAAT-3’ | Cloning *gshA* into pCDFTrc (Restriction site: BamHI-XhoI ) |
| gshA-pCDFTrc-R | 5’- GCGCTCGAGTCAGGCGTGTTTTTCCAGCC-3’ | Cloning *gshA* into pCDFTrc (Restriction site: BamHI-XhoI) |
| phoP-pCDFTrc-F | 5’-GCGGGATCCTAAGACAGGGAGAAATAAAAATGCG-3’ | Cloning *phoP* into pCDFTrc (Restriction site: BamHI-XhoI ) |
| phoP-pCDFTrc-R | 5’-GCGCTCGAGTCAGCGCAATTCGAACAGATAG-3’ | Cloning *phoP* into pCDFTrc (Restriction site: BamHI-XhoI ) |
| ypfN-pCDFTrc-F | 5’- GCGGGATCCTTCTGCAAGA GGAAATAAGC-3’ | Cloning *ypfN* into pCDFTrc (Restriction site: BamHI-XhoI ) |
| ypfN-pCDFTrc-R | 5’- GCGCTCGAGTTACTTCTTCGGTTGATCCTT-3’ | Cloning *ypfN* into pCDFTrc (Restriction site: BamHI-XhoI ) |
| gshA-pJN-F | 5’-GCGCCCGGGTTGACAGGCGGGAGGTCAAT-3’ | Cloning *gshA* into pJN105 (Restriction site: XmaI-XbaI) |
| gshA-pJN-R | 5’-GCGTCTAGATCAGGCGTGTTTTTCCAGCC-3’ | Cloning *gshA* into pJN105 (Restriction site: XmaI-XbaI) |
| phoP-pJN-F | 5’-GCGGAATTCTAAGACAGGGAGAAATAAAAATGCG-3’ | Cloning *phoP* into pJN105 (Restriction site: EcoRI-XbaI) |
| phoP-pJN-R | 5’-GCGTCTAGATCAGCGCAATTCGAACAGATAG-3’ | Cloning *phoP* into pJN105 (Restriction site: EcoRI-XbaI) |
| ypfN-pJN-F | 5’-GGGGAATTCTTCTGCAAGAGGAAATAAGC-3’ | Cloning *ypfN* into pJN105 (Restriction site: EcoRI-XbaI) |
| ypfN-pJN-R | 5’-GGGTCTAGATTACTTCTTCGGTTGATCCTT-3’ | Cloning *ypfN* into pJN105 (Restriction site: EcoRI-XbaI) |
| Pae-phoP-F | 5’-GGGGAATTCATCTCATCCGGAGGAACC-3’ | Cloning *phoP* derived from *P. aeruginosa* PA14 strain into pJN105 (Restriction site: EcoRI-XbaI) |
| Pae-phoP-R | 5’-GGGTCTAGATCACCGGCAGCGCTC-3’ | Cloning *phoP* derived from *P. aeruginosa* PA14 strain into pJN105 (Restriction site: EcoRI-XbaI) |
| PolyG-1st-1 | 5’-GTGACTGGAGTTCAGACGTGTGCTCTTCCGATCTGGGGGGGGGGGGGGGG-3’ | This study |
| Tn5-1st-1 | 5’-ACCTGCAGGCATGCAAGCTTCAGGG-3’ | This study |
| Tn5-2nd-1 | 5’-AATGATACGGCGACCACCGAGATCTACACTCTTTTCAGGGTTGAGATGTGTATAAGAGA-3’ | This study |

**Supplementary table 2.** List of genes involved in ampicillin resistance

| **Genes** | **Tn insertion ratio (after Amp selection/before Amp selection)** | **Expected Amp resistance phenotype^1^** | **Amp resistance phenotype in the absence of**  **β-lactamase^1,2^** | **Amp resistance phenotype in the presence of**  **β-lactamase^1,3^** | **Function** |
| --- | --- | --- | --- | --- | --- |
| *clpX* | 77.1 | R | R | R | ClpX ATP-dependent protease specificity component and chaperone |
| *clpP* | 207.8 | R | R | R | Serine protease |
| *xseA* | 0.01 | S | S | S | Exodeoxyribonuclease VII subunit XseA |
| *dedD* | 0.1 | S | S | S | Cell division protein |
| *yejG* | 0.01 | S | S | S | Function unknown |
| *dsbC* | 0.04 | S | S | S | Protein disulfide isomerase |
| *minC* | 0.04 | S | S | S | Z-ring positioning protein |
| *dhaL* | 0.05 | S | S | S | Dihydroxyacetone kinase subunit L |
| *pcnB* | 0.08 | S | S | S | poly(A) polymerase I |
| *rpoS* | 0.06 | S | S | S | RNA polymerase sigma factor |

^1^ ‘R’ indicates ampicillin resistant phenotype and ‘S’ indicates ampicillin sensitive phenotype, relative to the wild type strain.

^2, 3^ The deletion mutant of the respective gene was constructed and ampicillin resistance was tested by spotting assay as described in figure 1. ‘R’ indicates ampicillin resistant phenotype and ‘S’ indicates ampicillin sensitive phenotype, relative to the corresponding control strains.

**Supplementary table 3.** *E. coli* clinical isolate genomes with ampicillin phenotype

**Attached Excel File**

**Supplementary figures**


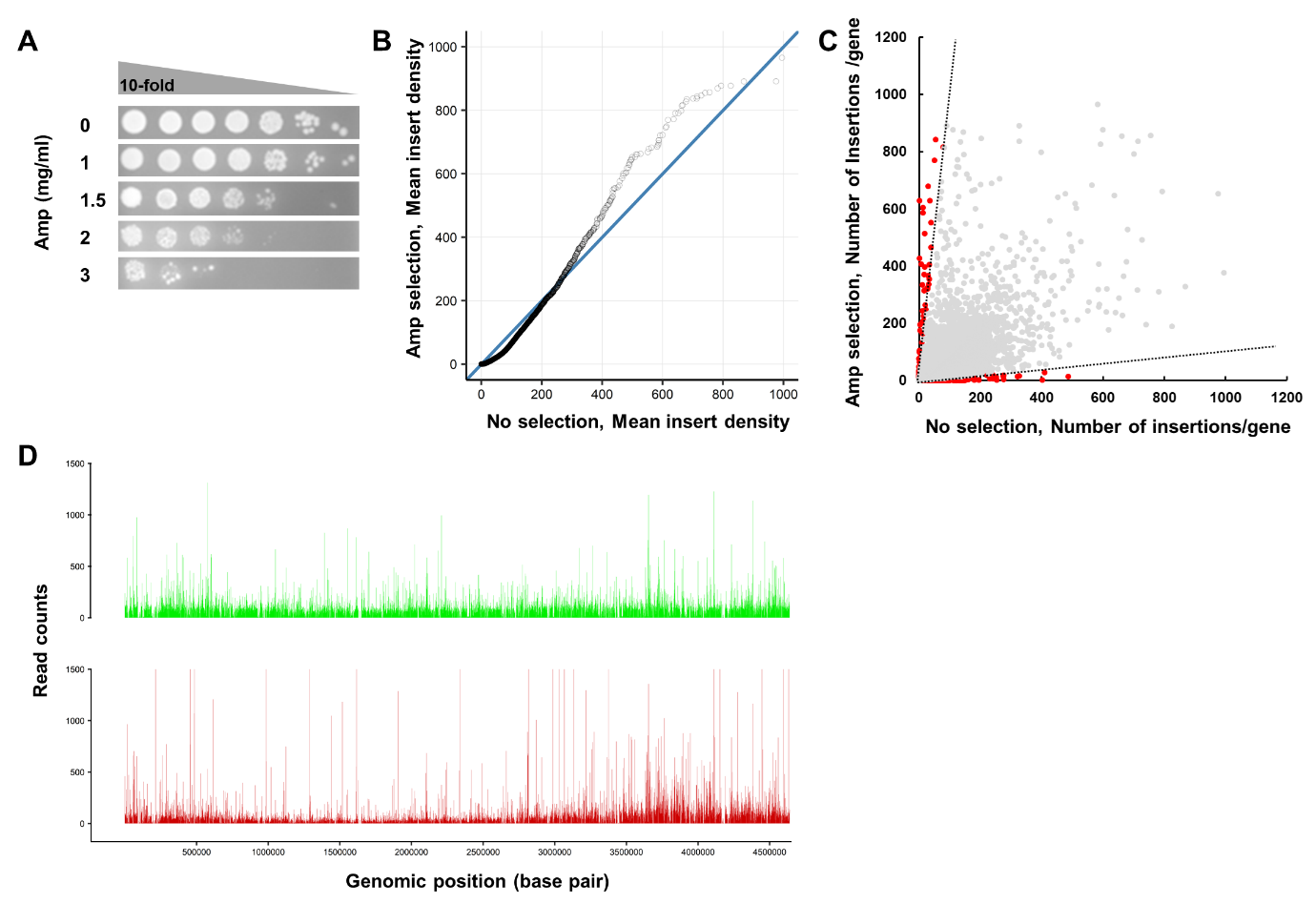


**Supplementary figure 1.** (A) The ampicillin (Amp) resistance was tested by spotting assay. MG1655 Δ*hsdR* pBR322 strain was examined for further Tn-Seq analysis. Under condition of 1.5 mg/ml of ampicillin, 10% of cells are viable and this concentration was chosen to be used for ampicillin selection. (B) The scatter Quantile-Quantile (Q-Q) plot demonstrates a positive linear correlation between the two variables, suggesting that as the mean insert density without selection increases, the mean insert density with ampicillin selection also increases. The data points closely align along the trend line, indicating a strong linear relationship without much deviation or outliers. This can suggest that ampicillin selection does not significantly alter the distribution of mean insert densities compared to no selection, implying a consistent effect of ampicillin across the range of insert densities observed. (C) The number of transposon insertions in each gene was compared between no selection and ampicillin selection conditions. Genes with more than a 10-fold difference between the two groups are shown in red. (D) Two comparative tracks of read counts across the genomic positions (base pairs) are shown. The top green track represents read counts from an experiment without selection pressure, while the bottom red track shows data from the ampicillin-selected condition, illustrating the distribution and frequency of transposon insertions throughout the genome. This uniform distribution suggests that the transposon insertions occur randomly and are not biased by genomic features or selection pressure.


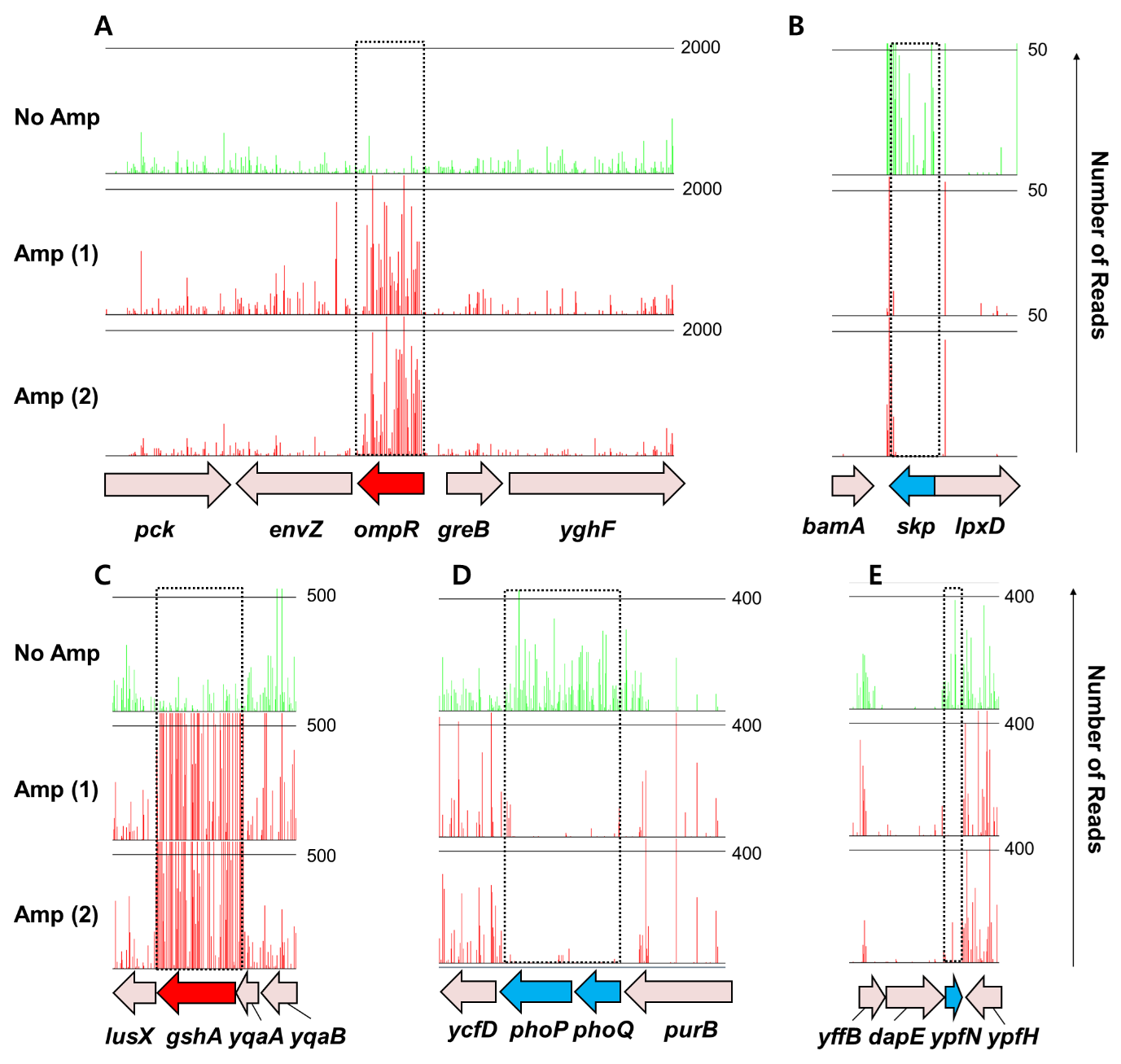


**Supplementary figure 2.** The reproducibility and consistency of transposon insertion patterns in a Tn-Seq experiment conducted with and without ampicillin (Amp) selection across multiple replicates. The top track (Green) shows the distribution of reads without ampicillin, and the subsequent tracks (Red) show the reads with ampicillin selection as replicates. The highlighted area indicates increased insertion frequency in the *ompR*, *skp*, *phoPQ* and *ypfN* (A-E).


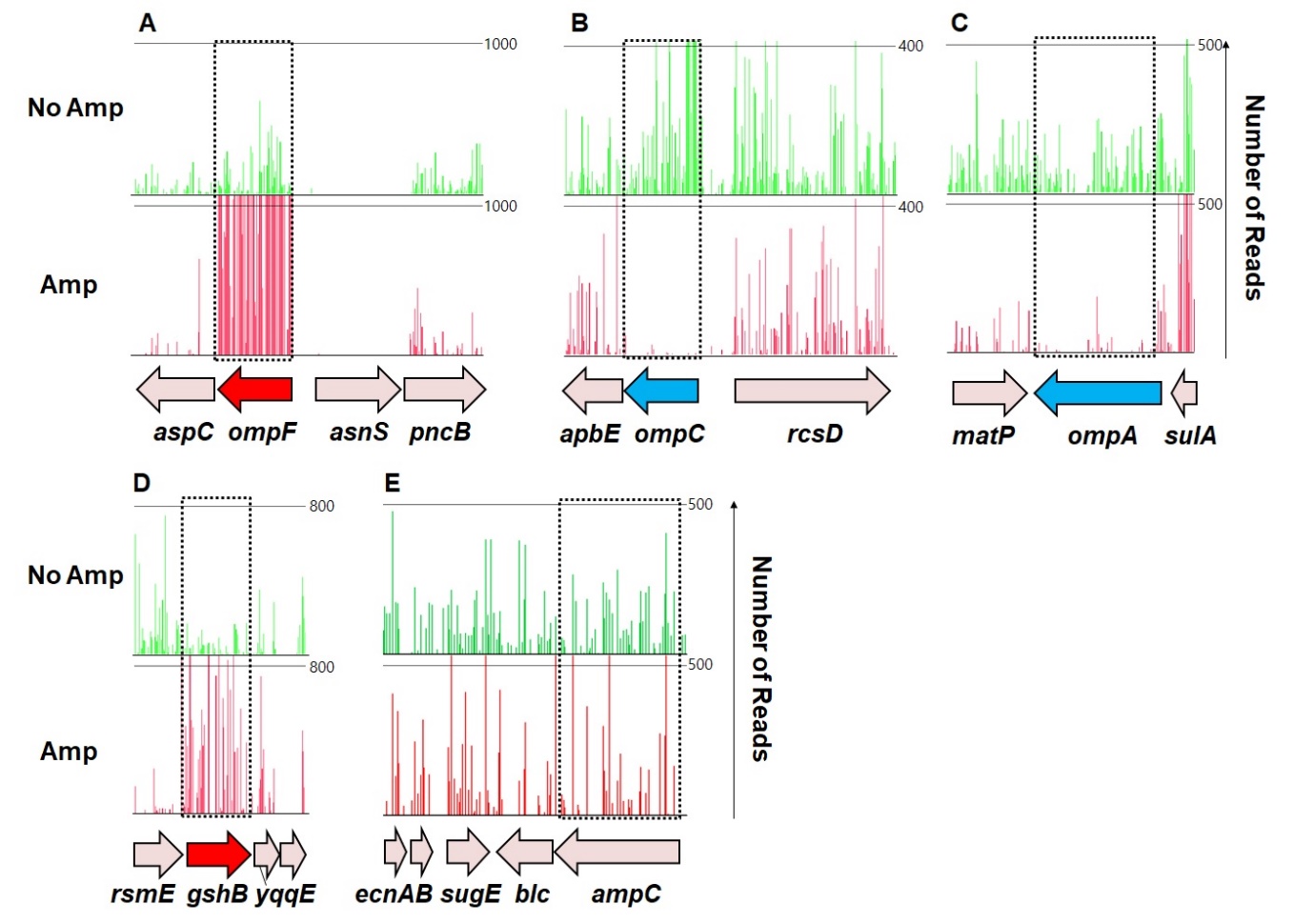


**Supplementary figure 3.** Transposon insertion frequency into the *ompF* (A), *ompC* (B), *ompA* (C), *gshB* (D) and *ampC* (E) genes. Transposon insertion frequency was increased 68-, 10-fold for *ompF* and *gshB* genes, respectively. *OmpC* and *ompA* genes showed decrease of transposon insertion frequency of 123-, and 8-fold, respectively. *AmpC* encodes β-lactamase, and the transposon insertion frequency was not altered, indicating that *ampC* is either not expressed or expressed at very low levels.


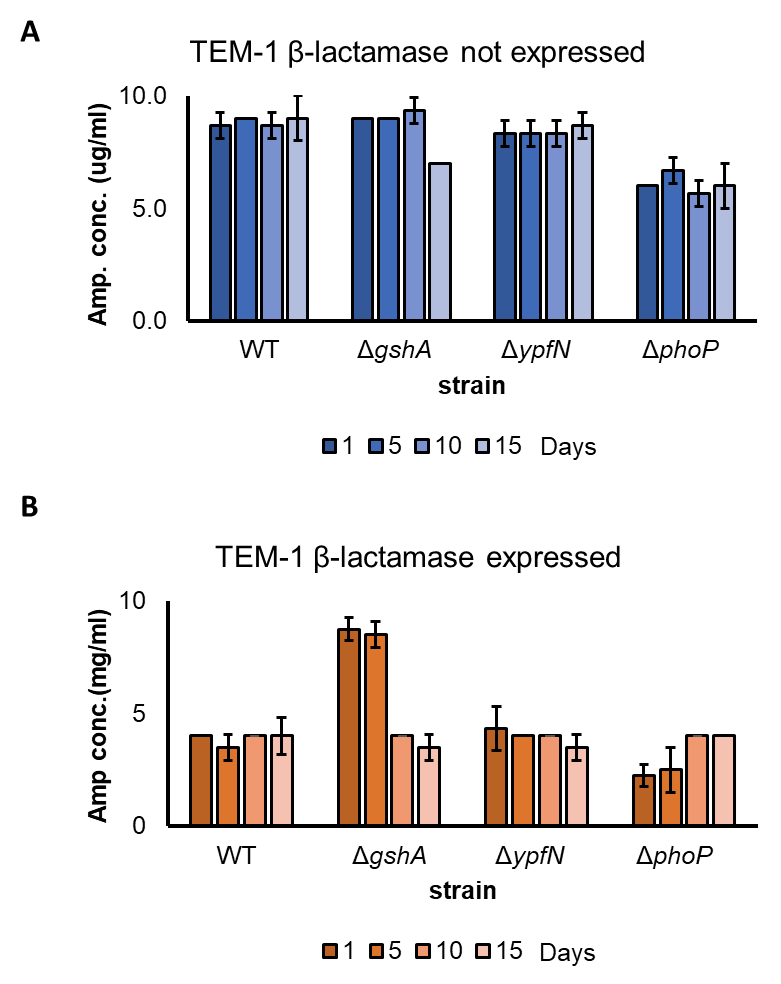


**Supplementary figure 4.** MIC shift assay demonstrates the stability of the ampicillin resistance phenotype over multiple passages. Wild-type and specified mutants were tested alone (A) and with β-lactamase expression (B). Passages were conducted for up to 15 days, and MIC values were measured at the corresponding time points.

**
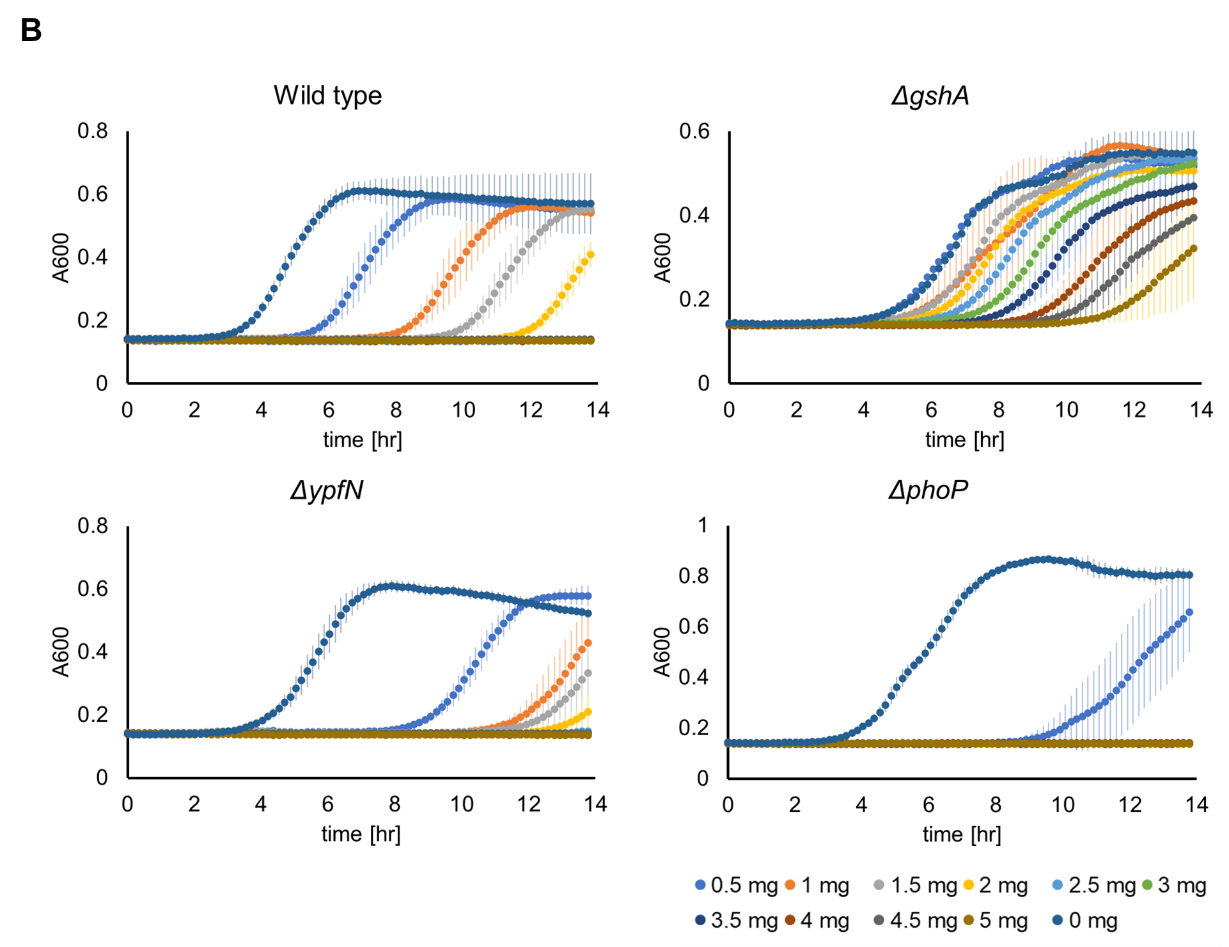

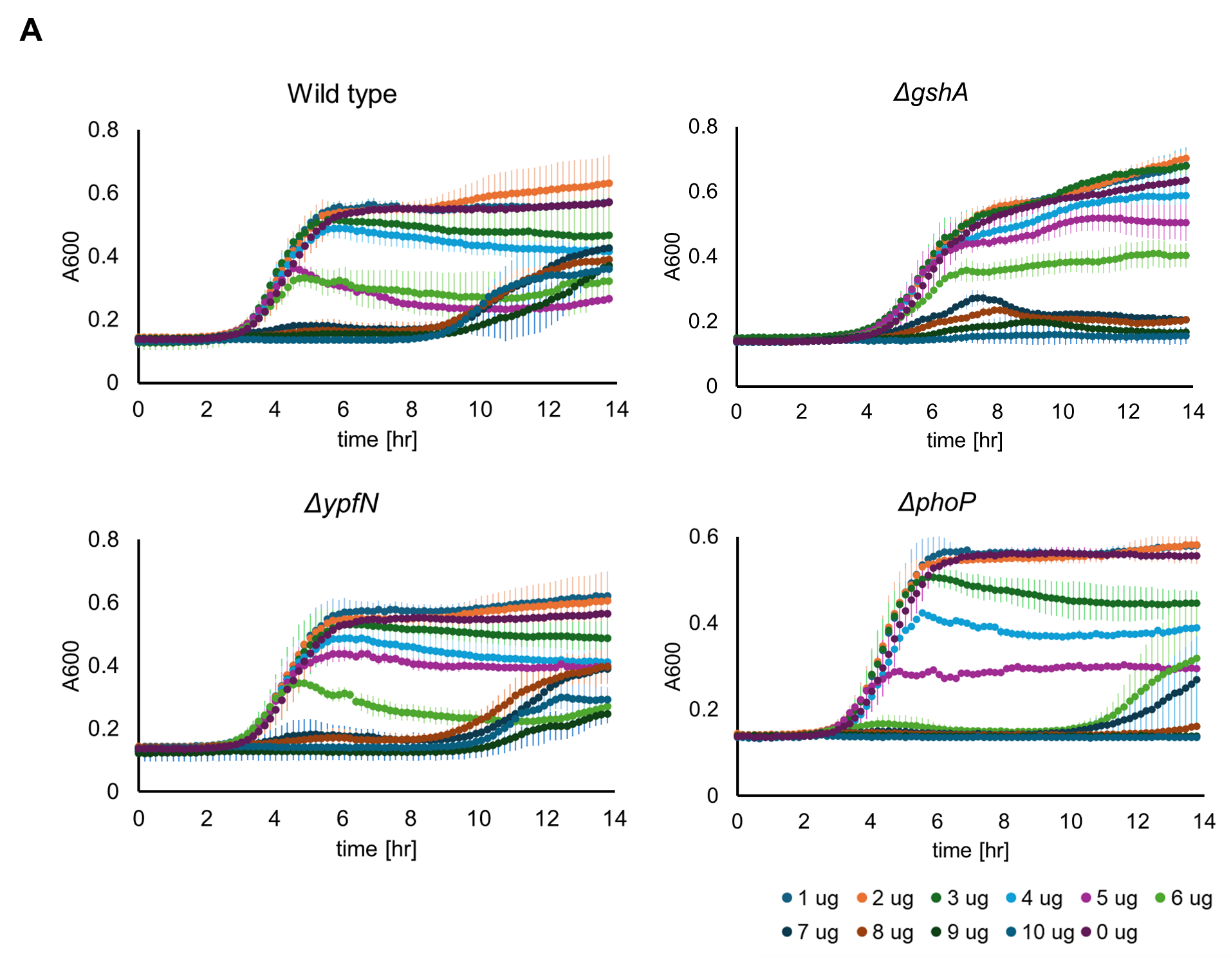
**

**Supplementary figure 5.** The cell growth curve was examined without TEM-1 β-lactamase expression (A) and with TEM-1 β-lactamase expression (B) in *E. coli* with various concentration of ampicillin in 200 μl of LB medium.


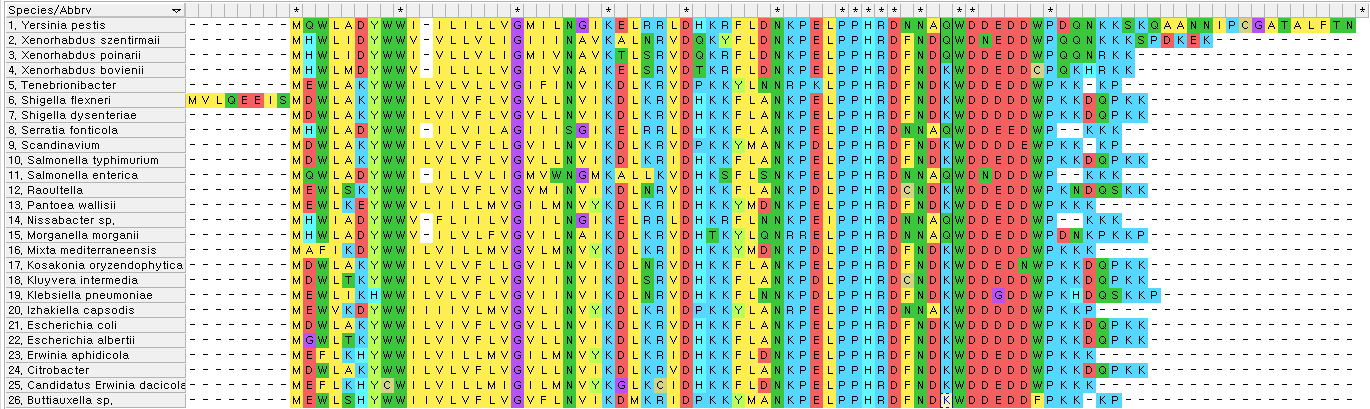


**Supplementary figure 6.** The amino acid sequences of YpfN homologous proteins were aligned by ClustalW and visualized by MEGA11 [[8](#_ENREF_8)]. The accession numbers for the sequences are as stated: *Escherichia coli*, Q2EET2; *Salmonella* Typhimurium, Q7CQ24; *Shigella dysenteriae*, Q32D97; *Shigella flexneri*, Q0T245; *Salmonella enterica*, A0A5Y3M7G4; *Yersinia pestis*, NSL64314.1; *Escherichia albertii*, WP_262938237.1; *Kosakonia oryzendophytica*, WP_088238188.1; *Citrobacter*, WP_012906607.1; *Raoultella*, WP_041146673.1; *Klebsiella pneumonia*, WP_255055298.1; *Buttiauxella sp.*, WP_139879504.1; *Erwinia aphidicola*, WP_048915230.1; *Candidatus Erwinia dacicola*, WP_070133962.1; *Tenebrionibacter*, WP_238713725.1; *Pantoea wallisii*, WP_128600035.1; *Xenorhabdus poinarii*, WP_045958380.1; *Xenorhabdus bovienii*, WP_071826701.1; *Serratia fonticola*, WP_142014649.1; *Nissabacter sp.*, WP_137762462.1; *Xenorhabdus szentirmaii*, WP_071991790.1; *Morganella morganii*, WP_046893236.1; *Kluyvera intermedia*, WP_234502355.1; *Mixta mediterraneensis*, WP_193404638.1; *Izhakiella capsodis*, WP_092876056.1; *Scandinavium*, WP_110509031.1.


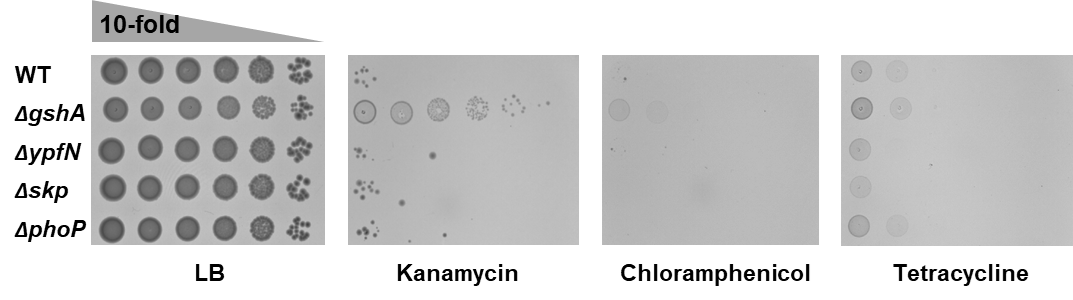
**Supplementary figure 7.** The antibiotic resistance of the deletion strains of *gshA*, *ypfN*, *skp* and *phoP* were texted. 5 μg/mL of kanamycin, 6 μg/mL of chloramphenicol and 1 μg/mL of tetracycline were used for spotting assay.


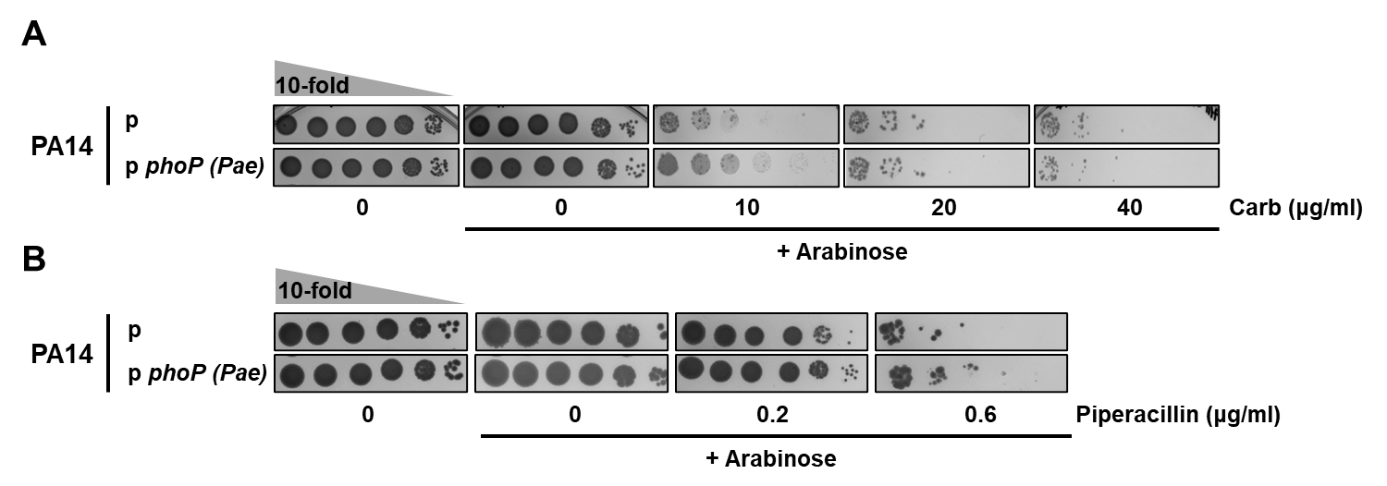


**Supplementary figure 8.** The *PhoP* gene, derived from the *P. aeruginosa* PA14 strain, was cloned into the pJN105 arabinose inducible plasmid and expressed in the *P. aeruginosa* PA14 strain. For the spotting assay, LB-agar medium supplemented with 1% arabinose and carbenicillin (Carb) concentrations of 10, 20, and 40 μg/mL (A) and 1% arabinose and piperacillin concentration of 0.2 and 0.6 μg/ml (B) were used.


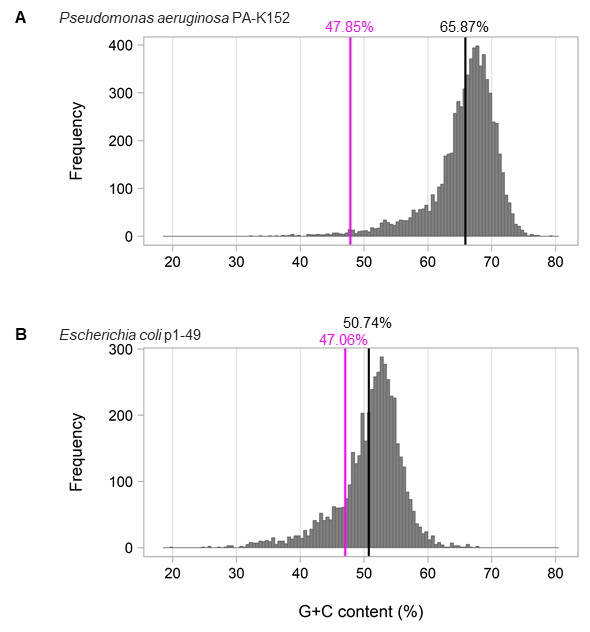


**Supplementary figure 9.** Histogram of G+C content distribution of genome-wide genes and YpfN-encoding gene (A) Analysis of *P. aeruginosa* strain PA-K152 (clinical isolate, accession number GCF_039413175.1). (B) Analysis of *E. coli* strain p1-49 (sputum isolate, accession number GCA_031584715.1). The genome-wide average G+C content is indicated with the black vertical line in each panel. The G+C content of the YpfN-encoding gene is indicated with the magenta vertical line in each panel.


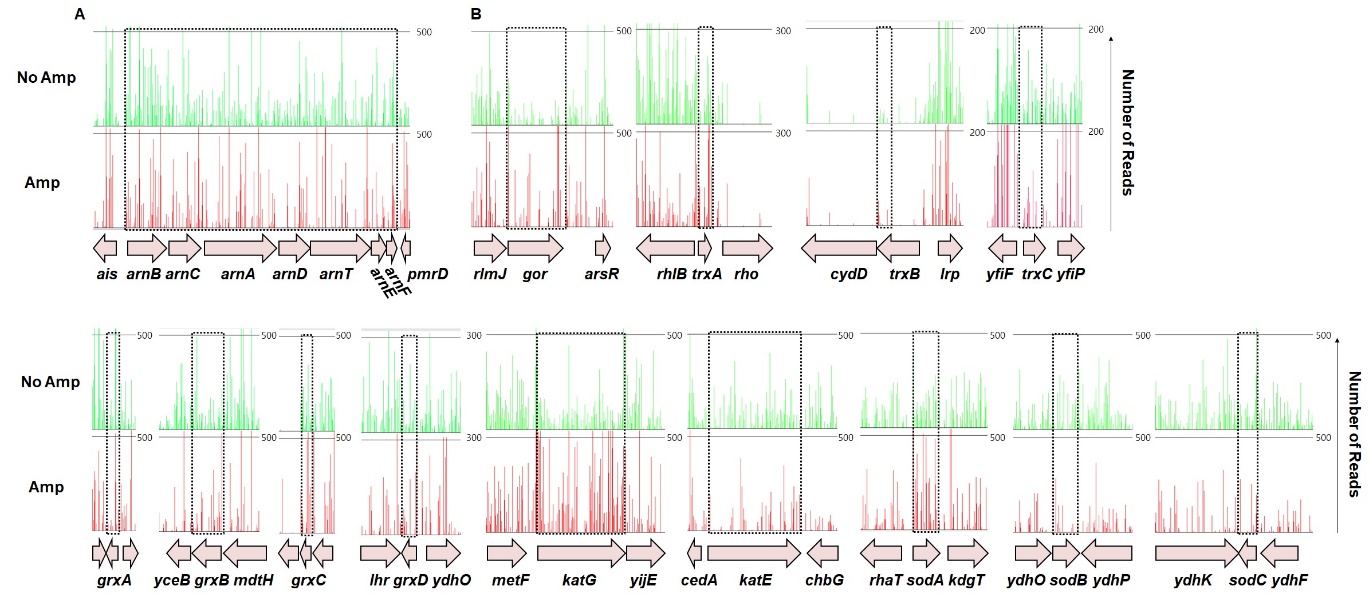


**Supplementary figure 10.** The Transposon insertion frequency into *arn* operon (A) and genes involved in response to reactive-oxygen species; *gor*, *trxA*, *trxB*, *trxC*, *grxA*, *grxB*, *grxC*, *grxD*, *katG*, *katE*, *sodA*, *sodB*, and *sodC* (B).


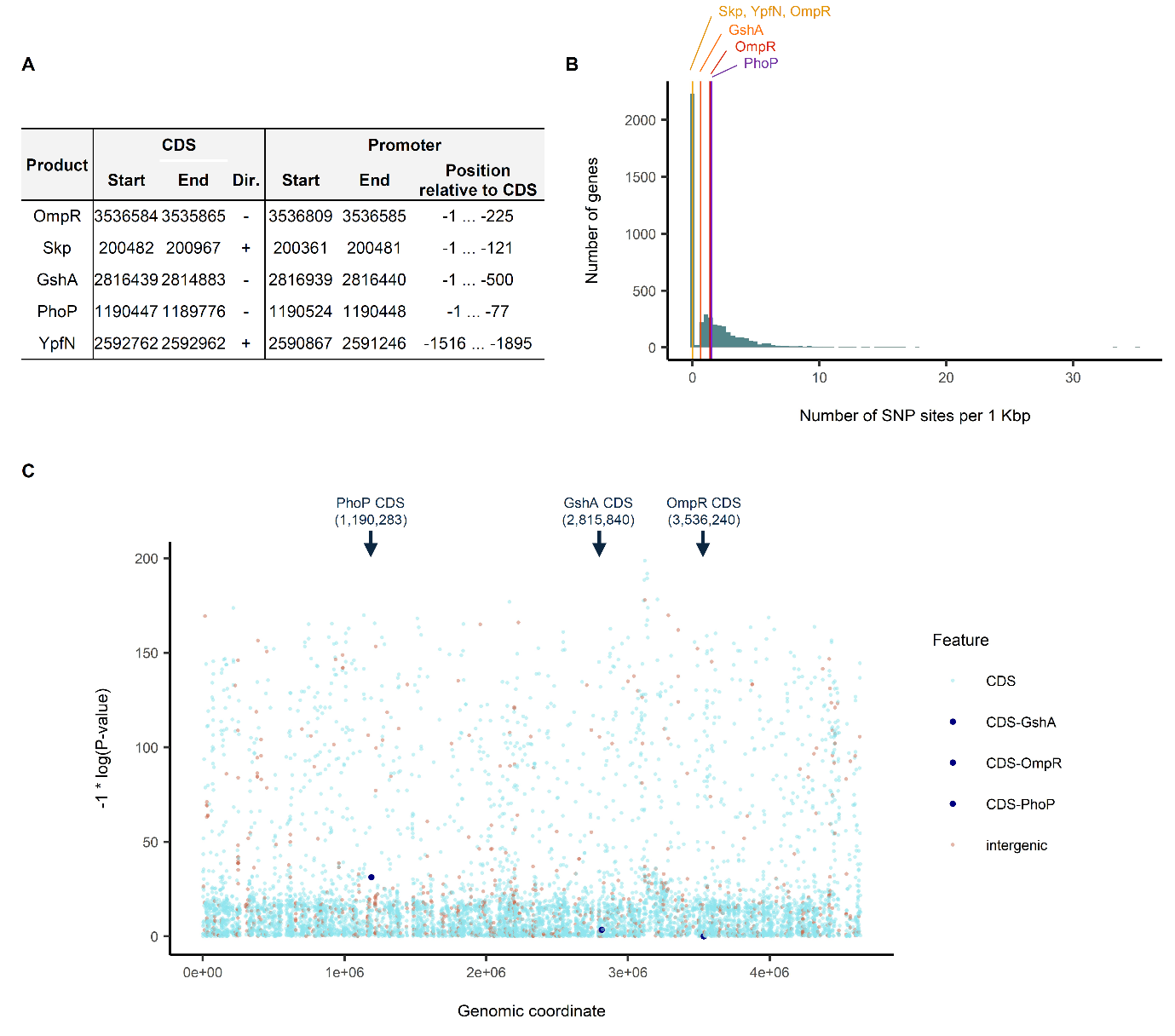


**Supplementary figure 11.** Genome-wide association analysis between the genome-wide SNPs (n = 7,268) and ampicillin phenotypes. (A) CDS and Promoter region of each gene on genome are listed (B) frequency of polymorphic sites in these genes (C) Genome-wide association analysis between the genome-wide SNPs (n = 7,268) and ampicillin phenotypes

**REFERENCES**

[1] Lai GC, Cho H, Bernhardt TG. The mecillinam resistome reveals a role for peptidoglycan endopeptidases in stimulating cell wall synthesis in *Escherichia coli*. PLoS genetics. 2017;13(7):e1006934.

[2] Langmead B. Aligning short sequencing reads with Bowtie. Current protocols in bioinformatics. 2010;32(1):11.7. 1-11.7. 14.

[3] Lennox E. Transduction of linked genetic characters of the host by bacteriophage P1. Virology. 1955;1(2):190-206.

[4] Baba T, Ara T, Hasegawa M, et al. Construction of *Escherichia coli* K‐12 in‐frame, single‐gene knockout mutants: the Keio collection. Molecular systems biology. 2006;2(1):2006.0008.

[5] Kadeřábková N, Mahmood AJ, Mavridou DA. Antibiotic susceptibility testing using minimum inhibitory concentration (MIC) assays. npj Antimicrobials and Resistance. 2024;2(1):37.

[6] Vinchhi R, Jena C, Matange N. Adaptive laboratory evolution of antimicrobial resistance in bacteria for genetic and phenotypic analyses. STAR protocols. 2023;4(1):102005.

[7] Sievers F, Higgins DG. Clustal Omega, accurate alignment of very large numbers of sequences. Multiple sequence alignment methods. 2014:105-116.

[8] Tamura K, Stecher G, Kumar S. MEGA11: molecular evolutionary genetics analysis version 11. Molecular biology and evolution. 2021;38(7):3022-3027.

[9] An S, Wu Je, Zhang L-H. Modulation of Pseudomonas aeruginosa biofilm dispersal by a cyclic-Di-GMP phosphodiesterase with a putative hypoxia-sensing domain. Applied and environmental microbiology. 2010;76(24):8160-8173.

[10] O'Toole GA. Microtiter dish biofilm formation assay. Journal of visualized experiments: JoVE. 2011 (47):2437.

[11] Hosseini A, Mas J. The β-galactosidase assay in perspective: Critical thoughts for biosensor development. Analytical Biochemistry. 2021;635:114446.

[12] Caiazza NC, Merritt JH, Brothers KM, et al. Inverse regulation of biofilm formation and swarming motility by *Pseudomonas aeruginosa* PA14. Journal of bacteriology. 2007;189(9):3603-3612.

[13] Abdelaziz AA, Kamer AMA, Al-Monofy KB, et al. Pseudomonas aeruginosa’s greenish-blue pigment pyocyanin: its production and biological activities. Microbial Cell Factories. 2023;22(1):110.

[14] Quan S, Koldewey P, Tapley T, et al. Genetic selection designed to stabilize proteins uncovers a chaperone called Spy. Nature structural & molecular biology. 2011;18(3):262-269.

[15] Choi E, Han Y, Park S, et al. A translation-aborting small open Reading frame in the intergenic region promotes translation of a Mg2+ transporter in *Salmonella typhimurium*. MBio. 2021;12(2):10.1128/mbio. 03376-20.

[16] Grace A, Sahu R, Owen DR, et al. *Pseudomonas aeruginosa* reference strains PAO1 and PA14: A genomic, phenotypic, and therapeutic review. Frontiers in Microbiology. 2022;13:1023523.

[17] Lee C, Peters V, Melefors Ö, et al. Draft genome sequence of *Pseudomonas aeruginosa* SG17M, an environmental isolate belonging to clone C, prevalent in patients and aquatic habitats. Genome announcements. 2014;2(2):10.1128/genomea. 00186-14.

[18] Lee C, Wigren E, Trček J, et al. A novel protein quality control mechanism contributes to heat shock resistance of worldwide‐distributed *Pseudomonas aeruginosa* clone C strains. Environmental microbiology. 2015;17(11):4511-4526.

[19] Kim ES, Bae H-W, Cho Y-H. A pilin region affecting host range of the *Pseudomonas aeruginosa* RNA phage, PP7. Frontiers in microbiology. 2018;9:247.

[20] Kim B-o, Jang H-J, Chung I-Y, et al. Nitrate respiration promotes polymyxin B resistance in *Pseudomonas aeruginosa*. Antioxidants & Redox Signaling. 2021;34(6):442-451.

[21] Balbás P, Soberón X, Merino E, et al. Plasmid vector pBR322 and its special-purpose derivatives—a review. Gene. 1986;50(1-3):3-40.

[22] Lennon CW, Thamsen M, Friman ET, et al. Folding optimization in vivo uncovers new chaperones. Journal of molecular biology. 2015;427(18):2983-2994.

[23] Newman JR, Fuqua C. Broad-host-range expression vectors that carry the L-arabinose-inducible *Escherichia coli* *araBAD* promoter and the araC regulator. Gene. 1999;227(2):197-203.
